# PCC0208025 (BMS202), a small molecule inhibitor of PD-L1, produces an antitumor effect in B16-F10 melanoma-bearing mice

**DOI:** 10.1371/journal.pone.0228339

**Published:** 2020-03-26

**Authors:** Zhengping Hu, Pengfei Yu, Guangying Du, Wenyan Wang, Haibo Zhu, Ning Li, Huijuan Zhao, Zhaoju Dong, Liang Ye, Jingwei Tian

**Affiliations:** 1 Medicine & Pharmacy Research Center, Binzhou Medical University, Yantai, Shandong, China; 2 School of Public Health and Management & Institute of Toxicology, Binzhou Medical University, Yantai, Shandong, China; 3 School of Pharmacy, Key Laboratory of Molecular Pharmacology and Drug Evaluation (Yantai University), Ministry of Education, Collaborative Innovation Center of Advanced Drug Delivery System and Biotech Drugs in Universities of Shandong, Yantai University, Yantai, China; Istituto Superiore di Sanità, ITALY

## Abstract

The increased PD-L1 expression induces poorer prognosis in melanoma. The small molecule inhibitors of PD-1/PD-L1 pathways have been an encouraging drug development strategy because of good affinity and oral bioavailability without immunogenicity and immunotoxicities of PD-1/PD-L1 antibodies. In this study, we studied the effects of PCC0208025 (BMS202), a small molecule inhibitor of PD-L1, on PD-1/PD-L1 binding and the cytokines secretion in human CD3^+^ cells *in vitro*. We also investigated the antitumor and immunomodulatory activity of PCC0208025 and the pharmacokinetics properties in B16-F10 melanoma-bearing mice. The results showed that PCC0208025 inhibited the PD-1/PD-L1 proteins binding, and rescued PD-L1-mediated inhibition of IFN-γ production in human CD3^+^ T cells *in vitro*. Furthermore, in B16-F10 melanoma-bearing mice, PCC0208025 presented the antitumor effects, enhanced IFN-γ levels in plasma, increased the frequency of CD3^+^CD8^+^ T and CD8^+^IFN-γ^+^ T and the ratios of CD8^+^/Treg, and deceased the CD4^+^CD25^+^CD127^low/−^ (Treg) number in tumor. Pharmacokinetics study found that PCC0208025 was absorbed and distributed into the tumors with much higher concentrations than those of the blockade against PD-1/PD-L1 binding. Our work suggests that PCC0208025 exhibited anti-tumor effects through inhibiting Treg expansion and increasing cytotoxic activity of tumor-infiltrating CD8^+^ T cells by the blockade of PD-1/PD-L1 binding, which may provide the pharmacological basis to develop small molecule inhibitors of PD-1/PD-L1 binding for PCC0208025 as a lead compound.

## Introduction

Melanoma is the main cancer which caused skin cancer-related deaths [1,2]. A 5-year survival rate is below 10% with traditional therapies [1–3]. Recently, immunotherapies have become the standard treatment regimens for the melanoma patients [2–4].

In the tumor microenvironment, the activated PD-1/B7-H1 (programmed cell death 1/PD-L1) signaling pathway makes T lymphocytes functionally inactivated [2,5], which decreases the anti-tumor activity of T cells. So far, the PD-1 antibodies (pembrolizumab and nivolumab), have been approved for the treatment of the advanced melanoma [2,6,7]. In addition, the PD-1 receptor ligand (PD-L1) antibodies (BMS-936559 and atezolizumab) have been studied for treating melanoma in preclinical mouse models and clinical trials [2,8].

However, the monoclonal antibodies has some disadvantages such as the immunogenicity, lower bioavailability, poor solid tumor tissue distribution and difficult controlled pharmacokinetics, and thus antibody related toxicities [9–13]. In contrast, the small molecules posses good affinity, specificity and oral bioavailability without the immunogenicity. The chemical inhibitors for targeting the PD-1/PD-L1 pathway or PD-1/PD-L1 interaction, such as small molecules, macrocyclic peptides, peptides and peptidomimetics, have been reported [9,14]. Several small molecule inhibitors of PD-L1 from Bristol-Myers Squibb (BMS) have been studied with the good blockade activity of PD-1/PD-L1 binding [10,13,15]. One of these agents, BMS-202 (N-(2-{[2-Methoxy-6-(2-methyl-biphenyl-3-ylmethoxy)-pyridin-3-ylmethyl]-amino}-ethyl)-acetamide), was resynthesized and renamed as PCC0208025 in our lab. BMS202 inhibits PD-1 and PD-L1 binding with an IC_50_ of 0.018 μM in an HTRF binding assay [13,16], which was superior to other BMS compounds according to the BMS patent (page 172) [16]. However, the patent did not include any additional biological data, and a thorough review of the literature revealed that no other in vitro and in vivo data has been reported for PCC0208025 (BMS202), indicating further activity validation in vivo and in vitro for PCC0208025 (BMS202) is warranted [9,13].

**Fig 1:**
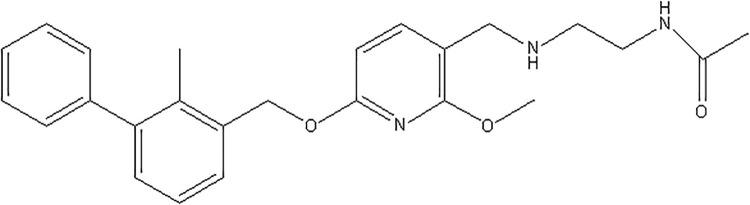
The structure of PCC0208025.

PCC0208025 is a PD-L1 inhibitor with a chemical structure as shown in Fig 1. In the present study, we investigated the potential effects of PCC0208025 on the PD-1/PD-L1 complex formation and the cytokines secretion in human CD3^+^ T cells *in vitro*. We also studied the antitumor and the immunomodulatory activity of PCC0208025, and pharmacokinetics properties in B16-F10 melanoma-bearing mice.

## Materials and methods

### Drug and reagents

PCC0208025 was synthetized as a white solid by biochempartner company (Shanghai, China) with the molecular formulae of C_25_H_29_N_3_O_3_. ^1^H NMR (300 MHz, DMSO-*d*_*6*_) δ 7.84 (s, 1H), 7.63 (d, *J* = 5.7 Hz, 1H), 7.47–7.36 (m, 4H), 7.31–7.17 (m, 4H), 6.43 (d, *J* = 6 Hz, 1H), 5.41 (s, 2H), 3.90 (s, 3H), 3.59 (s, 2H), 3.15–3.11 (m, 2H), 2.55 (t, 2H), 2.21 (s, 3H), 1.79 (s, 3H). MS (m/z) 420.3 [M+H]^+^. HPLC purity 98.62%.

The recombinant human PD-L1 protein (ab167713) was bought from Abcom company (USA) for studying the effects of PCC0208025 on IFN-γ secretion in human CD3^+^ cells *in vitro*. The human anti-PD-L1 antibody (BMS-936559) was expressed by Crown BioScience company (Suzhou, China). The anti-CD3^+^ antibody (MAB100, Clone # UCHT1) and anti-CD8^+^ antibody (MAB342-100, Clone # 37407) were provided by R&D Systems (USA). Human interferon gamma (IFN-γ) ELISA Kit (DIF50) and mice IFN-γ ELISA kit (MIF00) were purchased from R&D Systems (USA). Human PD-1/PD-L1 binding assay kit (Part # 64ICP01PEG) was obtained from Cisbio company (Shanghai, China), which mainly contains some reagents such as Tag2-PD-1, Tag1-PD-L1, anti-Tag1-EuK and anti-Tag2-XL665. BV421 anti-mouse CD3 (100228), BV510 anti-mouse CD4 (100449), FITC anti-mouse CD8 (100706), BV605 anti-mouse CD25 (102036) and APC anti-mouse CD127 (135012) were provided by BioLegend company (Canada). PE anti-mouse IFN-γ (12-7311-82) and the Intracellular Fixation & Permeabilization Buffer Set (88–8824) were obtained from Ebioscience company (USA). Leukocyte Activation Cocktail with GolgiPlug (550583) and FACS Staining Buffer (554656) was provided by BD Pharmingen Inc. (USA).

### Cytotoxicity of PCC0208025 to tumor cells and human CD3^+^ cells in vitro

Mouse melanoma cell line B16-F10 (ATCC^®^ Number: CRL-6475^™^) and mouse cell line CT26.WT (TCM37) were obtained from Cell Culture Center of the Institute of Basic Medical Sciences, Chinese Academy of Medical Sciences. The cells were maintained in DMEM supplemented with 10% (v/v) heat-inactivated fetal bovine serum (FBS) in a humidified 5% CO_2_ atmosphere at 37 °C. Tumor cells were plated in 96-well plate with a density of 5 × 10^3^ cells/well in 100 μL. After 24 h incubation, the cells were exposed to PCC0208025 with the final concentration of 2.5, 5, 10, 20, 40 an 80 μM in the final volume of 200 μL medium and cultured at 37 °C in a humidified atmosphere for 24 h and 48 h. 20 μL of 3-(4,5-dimethylthiazol-2-yl)-2,5-diphenyltetrazolium bromide (MTT, 5 mg/ml) was added to each well and incubated for additional 4 h at 37 °C. The medium was subsequently discarded, and 150 μL of DMSO was added to dissolve the formazan crystals [17]. The absorbance was measured at 570 nm using a Molecular Devices Spectra Max M5 (Molecular Devices, USA).

Human CD3^+^ cells with the purity above 95% were provided by LDEBIO company (Guangzhou, China). CD3^+^ cells were plated in the 96-well plate with a density of 1 × 10^5^ cells/well in 100 μL of DMEM medium containing 10% FBS. The 50 μL of anti-CD3 antibody and anti-CD28 antibody (aCD3 and aCD28, final concentration of 1 μg/mL for each) were added into 96-well plate. Then the CD3^+^ cells were exposed to PCC0208025 with the final concentration of 2.5, 5, 10, 20, 40 an 80 μM in the final volume of 200 μL medium, and cultured at 37 °C in a humidified atmosphere for 72 h. CellTiter-Glo (CTG) reagent was added into the wells to measure CD3^+^ cell viability.

### Effects of PCC0208025 on IFN-γ expression in human CD3^+^ cells in vitro

Human CD3^+^ cells were plated in the 96-well plate with a density of 1 × 10^5^ cells/well in 100 μL of DMEM containing 10% FBS. The 50 μL of aCD3 and aCD28 (final concentration of 1 μg/mL for each) were added into 96-well plate without or with 50 μL of human PD-L1 protein (final concentation of 10 nM). Then 50 μL of BMS-936559 or PCC0208025 solution with the final concentrations of 0.01, 0.1, 1 and 10 μM were added to the wells and cultured at 37 °C in a humidified atmosphere for 72 h. The supernatants were collected for detection of IFN-γ by using human IFN-γ ELISA Kit.

### Homogeneous Time-Resolved Fluorescence (HTRF) binding assay

In HTRF assay, the binding of Tag2-PD-1 and Tag1-PD-L1 is detected by anti-Tag1-EuK (HTRF donor) and anti-Tag2-XL665 (HTRF acceptor). Compounds or antibodies blocking PD-1/PD-L1 complex formation reduce the HTRF signal. Briefly, Tag2-PD-1 (20 nM final), PCC0208025 (0.15, 0.46, 1.37, 4.12, 12.35, 37.04, 111.11, 333.33 and 1000 nM, final) or BMS-936559 (0.002, 0.006, 0.024, 0.10, 0.39, 1.56, 6.25, 25 and 100 nM final), and Tag1-PD-L1 (2 nM final) were diluted in Binding Domain diluent buffer, and added one after the other to a 384-well plate for a total volume of 10 μl. After 15 min pre-incbation, an addition of 10 μl of detection reagent containing 1.83 nM anti-Tag1-EuK and 66.7 nM anti-Tag2-XL665 prepared in Binding Domain Detection buffer #1 was added into the assay well. The signals (665 nm/620 nm ratio) were obtained on Tecan M200 PRO. HTRF ratio = (OD_665 nm_/OD_620 nm_) × 10^4^.

### Animals

Male C57BL/6NCrl mice (4–5 weeks old; purchased from Vital River Laboratory Animal Technology Co., Ltd) were used for *in vivo* experiments. Animals were maintained under controlled environment at 25 °C on a 12-h light/dark cycle, which was free access to food and water. This experiment was approved by the Ethics Committee of Binzhou Medical University (No. 013 in 2014 for Animal Ethics Approval). The local legislation regarding the ethics of animal experimentation and the guidelines for the care and use of laboratory animals were followed in all animal procedures. All mice were intraperitoneally injected with 10 mg/kg of pentobarbital sodium to induce anesthesia before the surgery.

### In vivo tumor isograft model and dosing regimen

B16-F10 tumors were established by injecting 1 × 10^5^ cells mixed with matrigel into the dorsal area of male mice [18–20]. On 2^rd^ day, the mice bearing tumors were randomly divided into three groups (12/each group). Mice were administrated by oral gavage with PCC0208025 at 30 mg/kg or 60 mg/kg with a volume of 0.1 ml/10 g, twice daily. Control mice were given the same volume of saline. On days 7, 9, 11, 14, 16, 18 and 20, tumor dimensions were measured. Tumor volumes were calculated according to the following formula: volume (mm^3^) = 0.5 × length (mm) × width (mm) × width (mm). On day 20, all the mice were decapitated between 9:00 a.m. and 11:00 a.m.. The tumors were obtained. And the inhibition rate (IR) of tumor growth was calculated by the following formula: IR (%) = [(A − B)/A] × 100, where A and B were the mean tumor weight in the control and treatment groups, respectively.

### Measurements for plasma IFN-γ level in melanoma-bearing mice

Before all the mice were decapitated, the blood samples from orbital venous sinus were collected into tubes with heparin for plasma preparation. These samples were stored at -80°C for tests. Plasma IFN-γ level was determined by using mice ELISA kit according to the manufacturer’s instructions [21].

### Flow cytometry analyses for T lymphocytes in tumors from melanoma-bearing mice

At the end of the experiment (day 20), tumor tissues were harvested and 6 out of 12 were randomly selected according to the tumor weight in each group for flow cytometric analysis. Single cell suspensions were prepared and a Ficoll-Hypaque purification step was carried out for the tumor-derived cell suspension [22]. After the cells were washed twice with PBS and resuspended in DMEM supplemented with 1% FBS. 100 μL of cell suspension per tube, containing 2 × 10^5^ cells, was stimulated with 200 μL of Leukocyte Activation Cocktail with GolgiPlug in a 37°C humidified CO_2_ incubator for 6 h. Following activation, the cells were harvested and washed with FACS Staining Buffer, and used for antibody staining for 30 min at 4°C by using BV421 anti-mouse CD3, BV510 anti-mouse CD4, FITC anti-mouse CD8, BV605 anti-mouse CD25 and APC anti-mouse CD127. These tubes were centrifuged at 1200 rpm for 5 min and the supernatant were discarded, followed by an addition of 200 μl of the intracellular fixation buffer to each tube and incubating for 30 min at room temperature. The cells were washed twice with the permeabilization buffer and resuspended in the permeabilization buffer. PE anti-mouse IFN-γ antibody was added and incubated for 30 min in the dark at 4°C. The cell counts for the CD3^+^, CD3^+^CD4^+^, CD3^+^CD8^+^, CD4^+^CD25^+^CD127^low/−^ (Treg) and CD8^+^IFN-γ^+^ T lymphocytes were assessed via flow cytometry (BD FACSCanto II, California, USA). Finally, the ratios of CD8^+^/Treg were calculated.

### Pharmacokinetics of PCC0208025 in plasma and tumor from melanoma-bearing mice

In order to know the plasma and tumor concentrations of PCC0208025, 15 male C57BL/6NCrl mice were used to establish B16-F10 melanoma-bearing model according to the above method. When the tumors grew with the volume of about 1000 mm^3^, these mice were administrated by oral gavage with single dose of PCC0208025 at 60 mg/kg. At 1h, 3h and 8h after the dosing, 5 mice were decapitated, respectively, for collecting plasma and tumor tissues. All plasma samples were centrifuged for 10 min at 3000 g, separated and stored at -20 °C for followed analysis. The tumor tissues were homogenated in water (w/v = 1:4). The LC-MS/MS system consisted of an Agilent 1100 series HPLC system (Agilent Technologies, Waldbronn, USA) and a TSQ Quantum Assess tandem mass spectrometer (Thermo Electron Corporation, San Jose, CA, USA) equipped with ESI ion source operation in the positive mode. Data acquisition and processing were accomplished using the Xcaliber workstation. The chromatography separation was performed on a Waters symmetry CLU columns (150 × 2.1 mm i.e., 3.5 am, Warders, USA) with the flow rate of 0.2 ml/min in isocratic elution. The mobile phase consisted of acetonitrile-methanol-water (50:30:20, v/v/v) containing 0.1 mm ammonium acetate and 0.01% glacial acetic acid. The selective reactions monitoring mode was used to detect the analyses. The precursor/product transitions were at m/z 420.2–317.9 for PCC0208025, and m/z 427.1–207.0 for paliparidone (IS). The spray voltage was 4 kv. Sheath gas and auxilinry gas were 30 and 5 psi, respectively. The capillary temperature was 350 °C and argon gas pressure was 1.5 milli-Torr. The collision induced dissociation voltage was 15 V for PCC0208025 and 28 V for IS. The plasma and tumor drug concentration-time curves were displayed as PCC0208025 concentration in μM and μmol/kg, respectively, at various time points.

### Statistical analyses

The statistical analyses were performed using one-way ANOVA, followed by least significant difference (LSD) post hoc test in SPSS software (V 16.0). *P* < 0.05 was considered statistically significant.

## Results

### Cytotoxicity of PCC0208025 to tumor cells and human CD3^+^ cells in vitro

In order to investigate cytotoxicity of PCC0208025, both tumor cells and CD3^+^ cells were exposed to different concentrations of PCC0208025. The results in Table 1 showed that IC_50_ were above 10.0 μM to mice tumor cells and human CD3^+^ cells, which indicates PCC0208025 possesses low cytotoxicity *in vitro*.

**Table 1:** Effects of PCC0208025 on several cell lines viability were determined by MTT assay or CTG assay.

Time	IC_50_(μM)		
	B16F10	CT26	Human CD3^+^ cells
24 h	32.1 ± 2.3	21.9 ± 2.9	N/A
48 h	23.5 ± 3.2	15.3 ± 3.4	N/A
72 h	N/A	N/A	10.3 ± 1.3

N/A, not assay. IC_50_ values were calculated using Graph Pad Prism v 5.0. The results were presented as mean ± S.D (n = 3).

### HTRF binding assay

**Fig 2:**
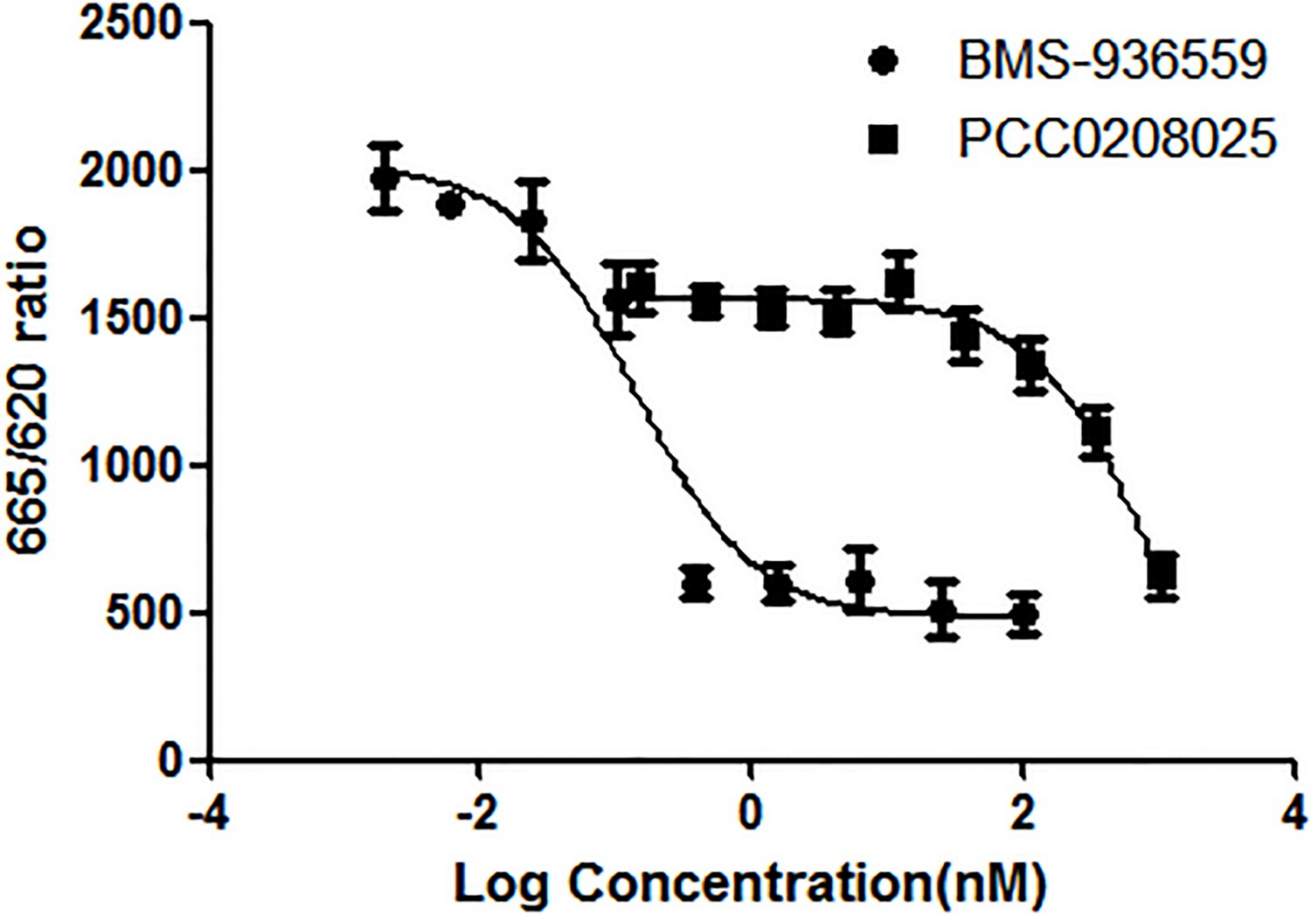
The inhibitory effects of PCC0208025 against PD-1 and PD-L1 binding. In HTRF assay, Tag2-PD-1 (20 nM final), PCC0208025 (0.15, 0.46, 1.37, 4.12, 12.35, 37.04, 111.11, 333.33 and 1000 nM, final) or BMS-936559 (0.002, 0.006, 0.024, 0.10, 0.39, 1.56, 6.25, 25 and 100 nM final), and Tag1-PD-L1 (2 nM final) were diluted in Binding Domain diluent buffer, and added one after the other to a 384-well plate for a total volume of 10 μl. After 15 min pre-incubation, an addition of 10 μl of detection reagent containing 1.83 nM anti-Tag1-EuK and 66.7 nM anti-Tag2-XL665 was added into the assay well. The signals (665 nm/620 nm ratio) were obtained on Tecan M200 PRO. HTRF ratio = (OD_665 nm_/OD_620 nm_) × 10^4^. The results showed that BMS-936559 and PCC0208025 presented high activity with IC_50_ of 0.54 nM and 235 nM.

In order to detect the inhibitory effects of PCC0208025 against PD-1 and PD-L1 binding, HTRF binding assay was conducted. Anti-PD-L1 antibody BMS-936559 and the compound PCC0208025 showed high activity with IC_50_ of 0.54 nM and 235 nM, respectively (Fig 2).

### Effects of PCC0208025 on IFN-γ level in human CD3^+^ cells in vitro

**Fig 3:**
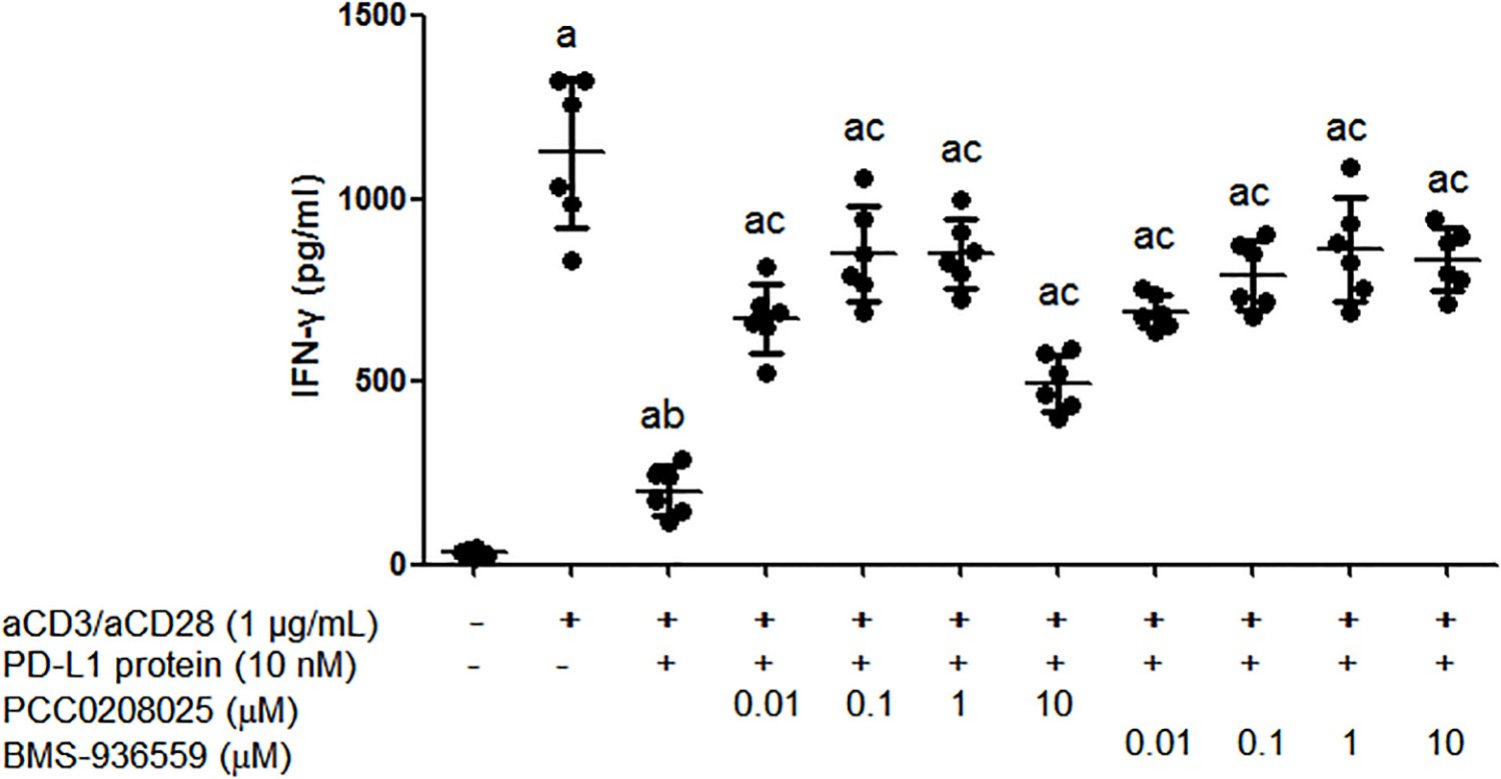
Effects of PCC0208025 on IFN-γ secreted by CD3^+^ cells *in vitro*. Human CD3^+^ cells were plated in the 96-well plate with a density of 1 × 10^5^ cells/well in 100 μL of DMEM containing 10% FBS. The 50 μL of aCD3 and aCD28 (final concentration of 1 μg/mL for each) were added into 96-well plate without or with 50 μL of human PD-L1 protein (final concentation of 10 nM). Then 50 μL of BMS-936559 or PCC0208025 solution with the final concentrations of 0.01, 0.1, 1 and 10 μM were added to the wells and cultured for 72 h. The supernatants were collected for detection of IFN-γ by using human IFN-γ ELISA Kit. The results were presented as mean ± SD (n = 6). ^a^*P* < 0.05 compared with medium control group. ^b^*P* < 0.05 compared with aCD3/aCD28 control group. ^c^*P* < 0.05 compared with aCD3/aCD28 and PD-L1 group.

Cytokines play an important role in the immune response, and we investigated the effects of PCC0208025 on the production of cytokine IFN-γ in human CD3^+^ cells *in vitro*. As shown in Fig 3, combined aCD3 and aCD28 significantly increased the IFN-γ expression compared with medium control (each treatment *P* < 0.05, n = 6), which was significantly decreased by human PD-L1 protein (each treatment *P* < 0.05, n = 6). However, anti-PD-L1 antibody BMS-936559 and the compound PCC0208025 from 0.01 to 1 μM markedly rescued PD-L1-mediated inhibition of IFN-γ production (each treatment *P* < 0.05, respectively, n = 6).

### Effects of PCC0208025 on the tumor growth in B16-F10-bearing mice

**Fig 4:**
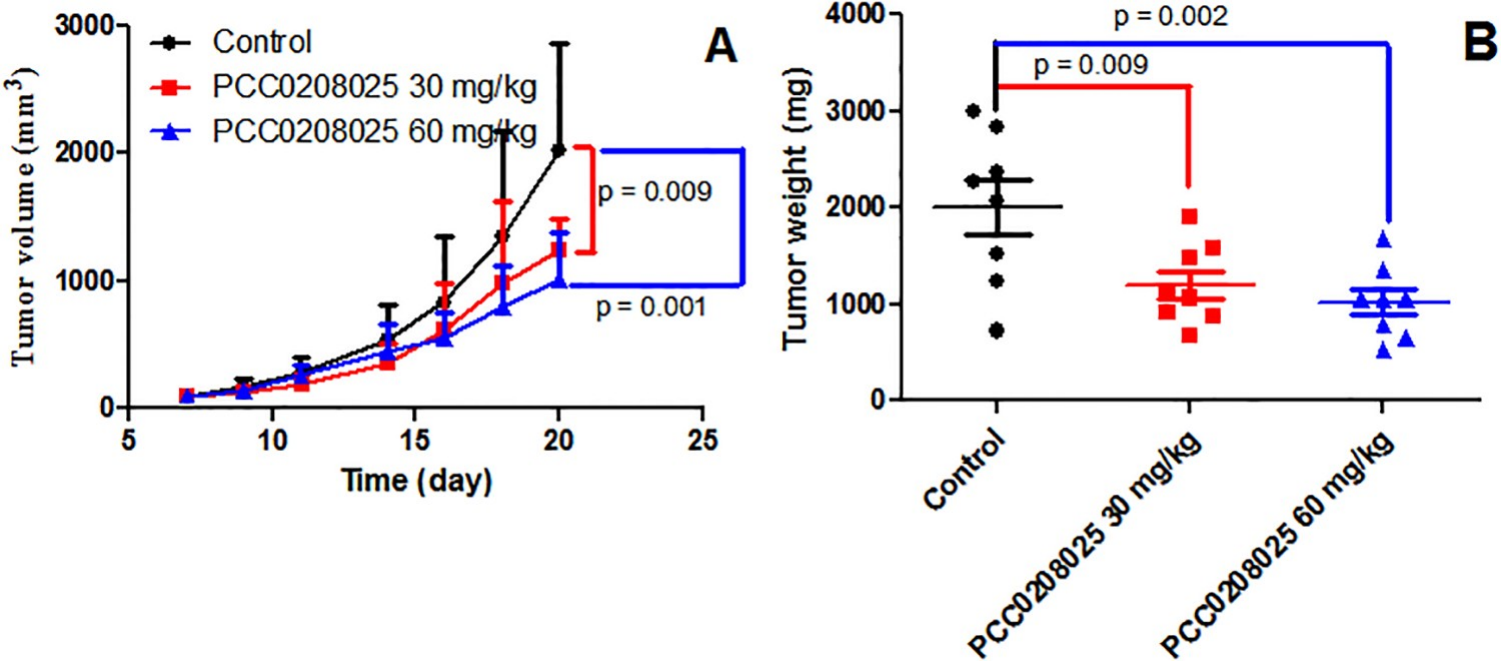
Effects of PCC0208025 on tumor growth in B16-F10 tumor isograft model. B16-F10 tumors were established by injecting 1 × 10^5^ cells mixed with matrigel into the dorsal area of male mice. On 2^rd^ day, the mice bearing tumors were randomly divided into three groups. Mice were administrated by oral gavage with PCC0208025 at 30 mg/kg or 60 mg/kg with a volume of 0.1 ml/10 g, twice daily. Control mice were given the same volume of saline. On days 7, 9, 11, 14, 16, 18 and 20, tumor dimensions were measured. On day 20, all the mice were decapitated between 9:00 a.m. and 11:00 a.m.. The tumors were obtained. And the inhibition rate (IR) of tumor growth was calculated. (A) PCC0208025 had tumor inhibition effects in tumor volume compared with control group (*P* < 0.05, n = 8) on day 20. (B) PCC0208025 had significant tumor inhibition effects in tumor weight compared with control group (*P* < 0.05, n = 8). The results were presented as mean ± SD. *P < 0.05 compared with control group.

We investigated the anti-cancer activities of PCC0208025 in B16-F10-bearing mice. We found that treatment with PCC0208025 at 30 mg/kg and 60 mg/kg significantly decreased tumor weight (*P* < 0.05, n = 8) and tumor volumes (day 20, *P* < 0.05, n = 8) compared with the control group (Fig 4A and 4B). According to tumor weight, 30 mg/kg and 60 mg/kg of PCC0208025 presented the IR of 30.3% and 50.1%, respectively.

### Effects of PCC0208025 on plasma IFN-γ level in melanoma-bearing mice

**Fig 5:**
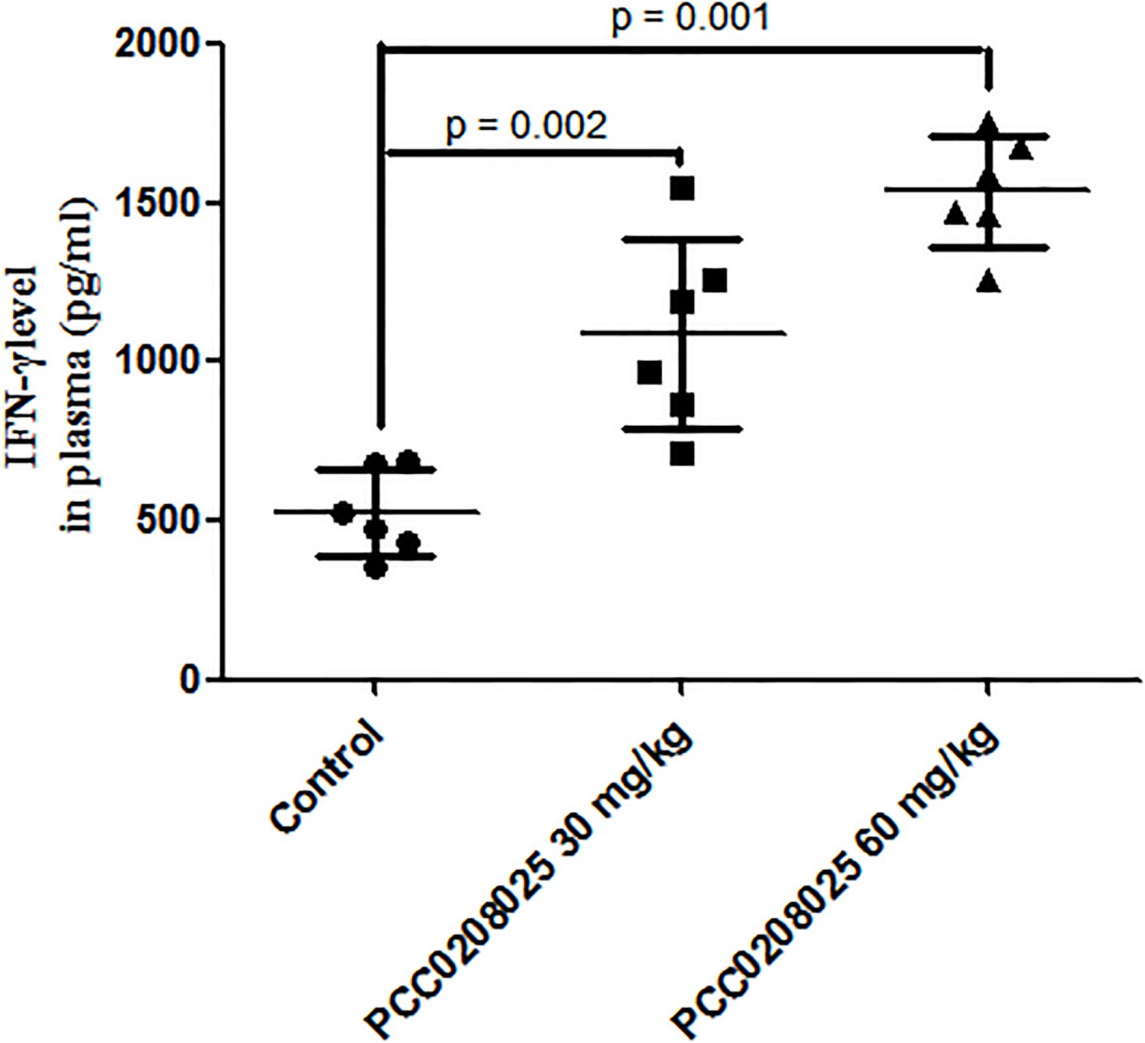
Effects of PCC0208025 on plasma IFN-γ level in B16-F10-bearing mice. Before all the mice with tumors were decapitated, the blood samples from orbital venous sinus were collected into tubes with heparin for plasma preparation. Plasma IFN-γ level was determined by using mice ELISA kit. The increase in plasma IFN-γ levels was observed in 30 mg/kg and 60 mg/kg groups compared with the control group (each treatment *P* < 0.05, respectively, n = 6). The results were presented as mean ± SD. *P < 0.05, compared with control group.

To investigate the effects of PCC0208025 on the immune function in B16-F10-bearing mice, we detected the plasma IFN-γ level using mice ELISA kit. As shown in Fig 5, PCC0208025 of 30 and 60 mg/kg markedly elevated plasma IFN-γ levels compared with the control group (each treatment *P* < 0.05, n = 6).

### Flow cytometry analyses for T lymphocytes in tumors

**Fig 6:**
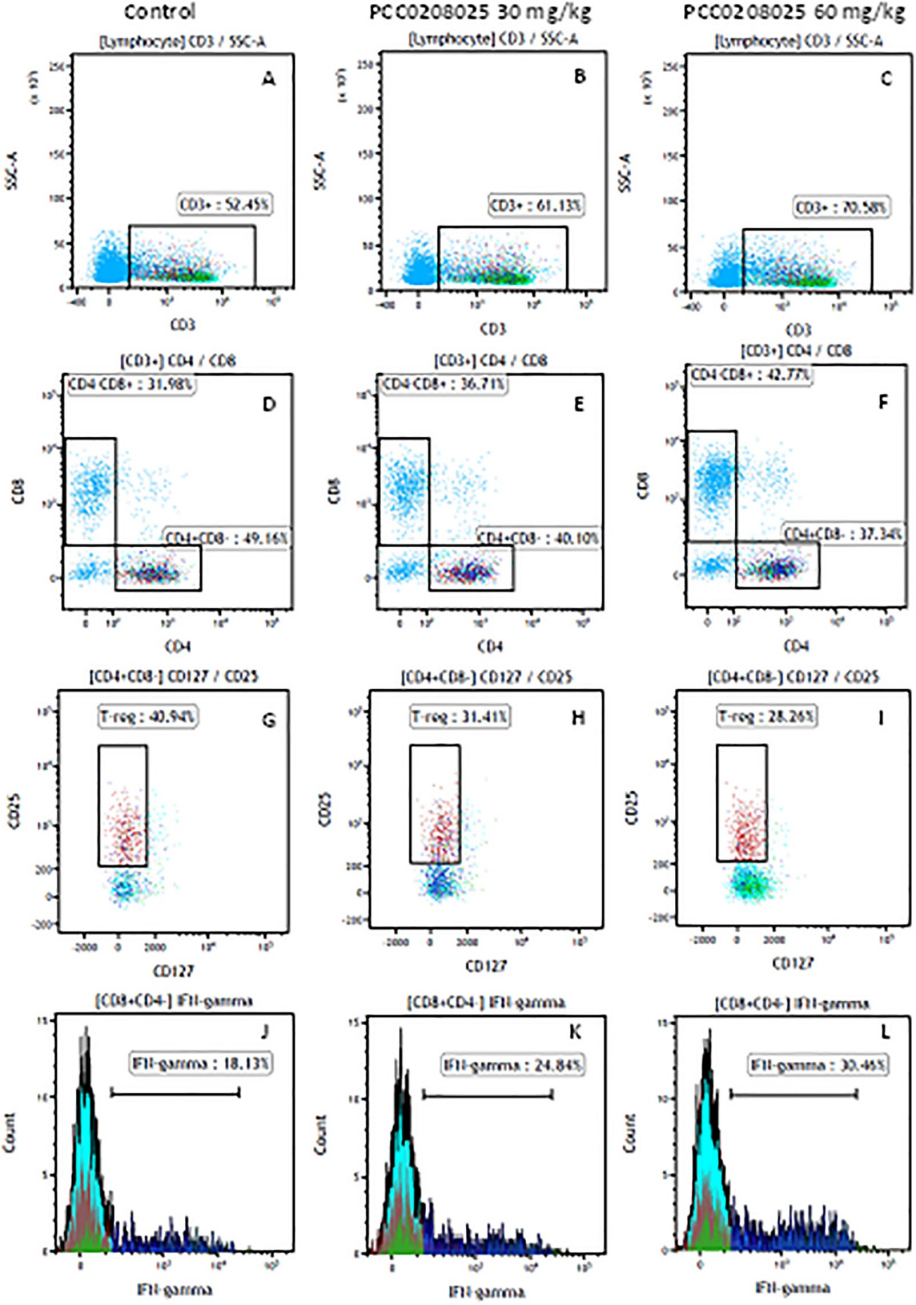
The representative figures for T cell subsets counted by flow cytometry from tumors in B16-F10-bearing mice. The cell counts for CD3^+^ (A, B and C), CD3^+^CD4^+^ (D, E and F), CD3^+^CD8^+^ (D, E and F), CD4^+^CD25^+^CD127^low/-^ (G, H and I) and CD8^+^IFN-γ^+^ (J, K and L) T lymphocytes from mouse tumor were determined by flow cytometry.

**Fig 7:**
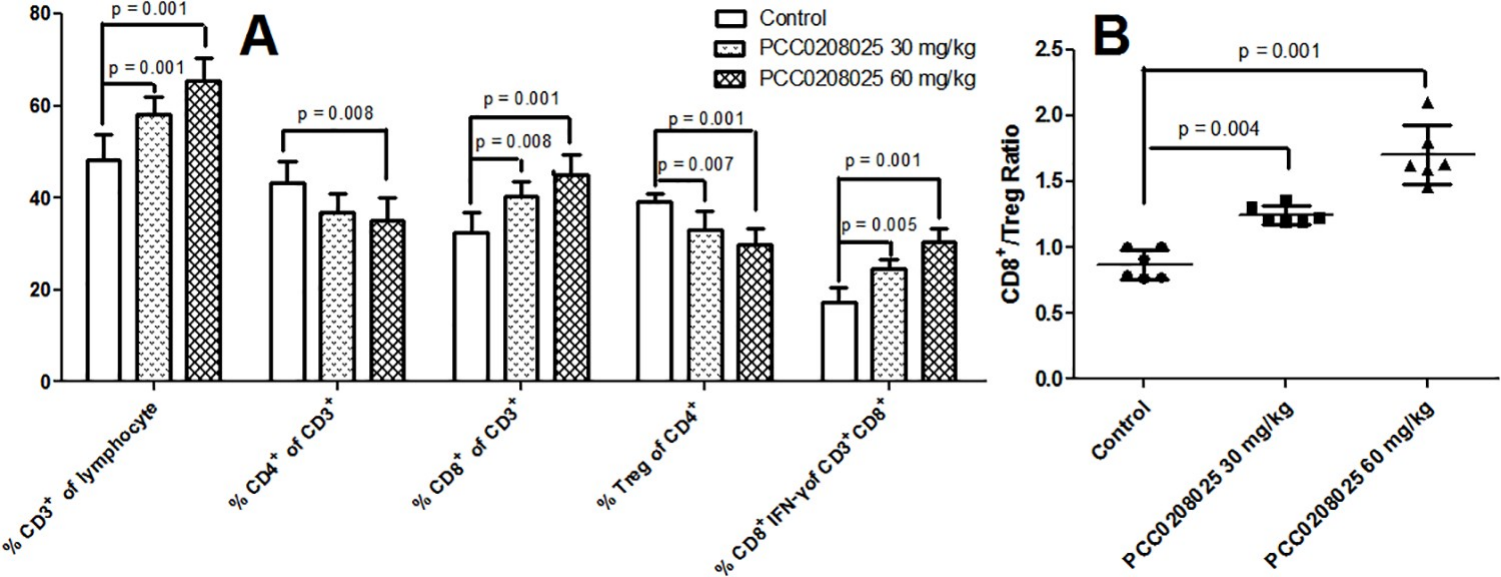
Effects of PCC0208025 on T cell subsets from tumor in B16-F10-bearing mice. The percentage of CD3^+^, CD3^+^CD8^+^ or CD8^+^IFN-γ^+^ T cells was markedly increased in PCC0208025 groups compared with the control group (each treatment *P* < 0.05 for each T cell, respectively, n = 6) (A). And, The significant decreases in the percentage of CD3^+^CD4^+^ T cells were observed in PCC0208025 60 mg/kg group compared with the control group (*P* < 0.05, n = 6) (A). However, the percentage of CD4^+^CD25^+^CD127^low/-^(Treg) was significantly decreased by 30 mg/kg and 60 mg/kg of PCC0208025 compared with the control group (each treatment *P* < 0.05, respectively, n = 6) (A). Furthermore, in PCC0208025 30 mg/kg and 60 mg/kg groups, the ratios of CD8^+^/Treg (B) were significantly increased compared with the control group (each treatment *P* < 0.05, respectively, n = 6). The results were presented as mean ± SD. *P < 0.05 compared with control group.

To investigate the effects of PCC0208025 on the cellular immunity, the cell counts for CD3^+^, CD3^+^CD4^+^, CD3^+^CD8^+^, CD4^+^CD25^+^CD127^low/−^ and CD8^+^IFN-γ^+^ T lymphocytes in tumor were determined by flow cytometry. As shown in Figs 6 and 7, the percentage of CD3^+^, CD3^+^CD8^+^ or CD8^+^IFN-γ^+^ T cells was markedly increased in PCC0208025 treatment groups compared with the control group (each treatment *P* < 0.05 for each T cell, respectively, n = 6). And, significant decreases in the percentage of CD3^+^CD4^+^ T cells were observed in PCC0208025 60 mg/kg group compared with the control group (*P* < 0.05, n = 6). However, the percentage of CD4^+^CD25^+^CD127^low/−^ (Treg) was significantly decreased by 30 mg/kg and 60 mg/kg of PCC0208025 compared with the control group (each treatment *P* < 0.05, respectively, n = 6). Furthermore, in PCC0208025 30 mg/kg and 60 mg/kg groups, the ratios of CD8^+^/Treg were significantly increased compared with the control group (each treatment *P* < 0.05, respectively, n = 6).

### Pharmacokinetics of PCC0208025 in plasma and tumor from melanoma-bearing mice

**Fig 8:**
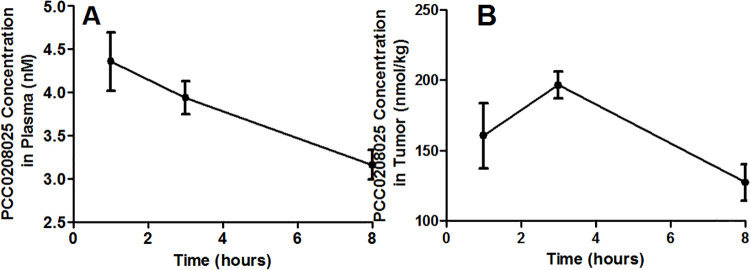
Pharmacokinetics of PCC0208025 in plasma and tumors in melanoma-bearing mice. After a single dose of 60 mg/kg PCC0208025, average concentrations were about 4.36, 3.94 and 3.16 nM in plasma at 1h, 3h and 8h, respectively (each time point, n = 5); and about 160.7, 196.7 and 127.3 nmol/kg in tumor at 1h, 3h and 8h, respectively (each time point, n = 5).

For determining PCC0208025 pharmacokinetics characteristics in melanoma-bearing mice, the plasma and tumor PCC0208025 concentrations were detected by HPLC-MS. After a single dose of 60 mg/kg, average concentrations were about 4.36, 3.94 and 3.16 nM in plasma at 1h, 3h and 8h, respectively (each time point, n = 5); and about 160.7, 196.7 and 127.3 nmol/kg in tumor at 1h, 3h and 8h, respectively (each time point, n = 5). These data showed PCC0208025 concentrations decreased slowly in plasma and tumor, while tumor tissue obtained higher concentration at 3h (Fig 8).

## Discussion

BMS recently disclosed the first non-peptidic small molecule inhibitors against the PD-1/PD-L1 pathway that highlighted the activity in a HTRF binding assay, including PCC0208025 (BMS-202) [16]. Udhwani, T reevaluated 311 PD-L1 ligand inhibitors and the docking results showed that BMS-202 still bind to PD-L1 dimeric structure with the highest affinity [14]. Because of the potential of BMS-202 (PCC0208025) as the lead compound of PD-L1 inhibitor, we further evaluated PCC0208025.

In out study, we found that PCC0208025 neither directly inhibit B16-F10 and CT-26 cells growth *in vitro*, nor killed human CD3^+^ cells at the designed concentration. However, PCC0208025 significantly decreased tumor volumes and tumor weights in B16-F10-bearing mice. These above results demonstrated that PCC0208025 did not directly kill tumor cells, which promoted us to carry out the further experiments to investigate the antitumor mechanisms in melanoma.

The PD-1 and PD-L1 interactions play a critical role in the tumor immune escape by inhibiting effector T cell proliferation, decreasing cytotoxic activity, inducing apoptosis in tumor-infiltrating T cells, and increasing Treg expansion [23]. In our study, HTRF binding assay showed that PCC0208025 presented the inhibition activity against the PD-1 and PD-L1 binding with IC_50_ of 235 nM, which is lower than the results obtained in the literature [13,14]. The different results were due to different HTRF kits. Furthermore, this report proved that PCC0208025 can occlude the PD-1 interaction surface of PD-L1 to form a dimeric protein complex [13,14], which provides a rationale that PCC0208025 can inhibit the PD-1/PD-L1 signaling pathway in T cells.

In order to evaluate the effects of PCC0208025 on the PD-1/PD-L1 signaling pathway, we investigated the rescue effects of PCC0208025 on the IFN-γ production decrease induced by human PD-L1 protein in human CD3^+^ T cells *in vitro*. We found that PCC0208025 rescued PD-L1-mediated inhibition of IFN-γ production, which was due to the blockade of PCC0208025 against PD-1/PD-L1 signaling pathway.

The T cells are very important for inducing the immune response to tumor antigens [2]. Our study showed that less CD3^+^CD8^+^ T cells were presented in the untreated B16-F10 tumor, which was closely related with tumor growth. As the result of the PD-1/PD-L1 signaling pathway blockade, PCC0208025 increased the CD3^+^CD8^+^ T cells frequency and T cells infiltration into tumor, which led to tumor growth inhibition.

A series of cytokines, such as IL-2, IFN-γ and TNF-α, can promote the CD8^+^ cytotoxic T cells production [2]. The activated CD8^+^ T cells into the tumor microenvironment can secrete the cytokine IFN-γ, which is closely related with the antitumor activity [24]. In our study, the results showed that PCC0208025 not only increased the IFN-γ level in plasma, but also increased the number of CD8^+^IFN-γ^+^ T cells in tumors in B16-F10 melanoma-bearing mice, which either induce immune cells proliferation and differentiation or produce direct tumor-killing effects to inhibit tumor growth [24].

In tumors, Treg are viewed as anti-tumor suppressors, and can decrease anti-tumor immune responses [25]. Most Treg cells are defined based on CD4, CD25 and FOXP3 expression. However, FOXP3 has a limited expression in Treg cells [25]. Recently, low level of CD127 on Treg cell surface are found, which is inversely correlated with FOXP3 level [25,26]. Thus, CD4^+^CD25^+^CD127^low/−^ can be the more reliable biomarker of Treg cells. PD-1 antibodies can decrease the Treg level and prevent Treg-mediated inhibition of cytotoxic T lymphocytes [23,25,27]. In our study, PCC0208025 not only decreased the percentage of Treg (CD4^+^CD25^+^CD127^low/−^ T cells), but also increased the ratios of CD8^+^/Treg in tumor. These results indicated that PCC0208025 inhibited Treg expansion and increased cytotoxic activity of tumor-infiltrating CD8^+^ T cells by the blockade of PD-1/PD-L1 binding.

Our pharmacokinetics study found that the average concentrations of PCC0208025 in plasma and tumor, at 1h, 3h and 8h after B16-F10 tumor-bearing mice were administrated with 60 mg/kg of PCC0208025, were similar to the IC_50_ values of PCC0208025 blockade against PD-1 and PD-L1 binding. These results indicated that PCC0208025 can be easily absorbed and distributed into the tumors to obtain the higher concentrations for producing the blockade effects against PD-1 and PD-L1 binding.

In summary, the PD-1/PD-L1 antibodies have already widely used for treatment of melanoma. However, these antibodies have some disadvantages such as the immunogenicity and immunotoxicities. In contrast, the small molecule compounds have good oral bioavailability without the immunogenicity and immunotoxicities. The small molecule interaction “hot spots” on PD-L1 surfaces suggest the approaches for the PD-1/PD-L1 antagonist drug discovery [13], which triggers further activities to discover small molecule inhibitors for PD-1/PD-L1 interactions with pharmacological effects. Our work suggests that PCC0208025 exhibited anti-tumor effects in B16-F10 tumor isograft model through inhibiting Treg expansion and increasing cytotoxic activity of tumor-infiltrating CD8^+^ T cells by the blockade of PD-1/PD-L1 binding, which provides the pharmacological basis to develop small molecule inhibitors for PD-1/PD-L1 interactions for PCC0208025 as a lead compound.

## Supporting information

S1 Fig LC-MS analysis from compound PCC0208025. (PDF)

S2 Fig The 1H spectra data of compound PCC0208025. (PDF)

S3 Fig Flow cytometry plots for each group. (PDF)

S1 Table The inhibitory effects of PCC0208025 against PD-1 and PD-L1 binding. In HTRF assay, Tag2-PD-1 (20 nM final), PCC0208025 (0.15, 0.46, 1.37, 4.12, 12.35, 37.04, 111.11, 333.33 and 1000 nM, final) or BMS-936559 (0.002, 0.006, 0.024, 0.10, 0.39, 1.56, 6.25, 25 and 100 nM final), and Tag1-PD-L1 (2 nM final) were designed. After pre-incubation, anti-Tag1-EuK and anti-Tag2-XL665 was added into the assay well. The signals (665 nm/620 nm ratio) were obtained on Tecan M200 PRO. HTRF ratio = (OD665 nm/OD620 nm) × 10^4^.
(DOCX)

S2 Table Effects of PCC0208025 on IFN-γ secreted by CD3^+^ cells *in vitro*. Human CD3^+^ cells were plated in the 96-well plate with a density of 1 × 10^5^ cells/well in 100 μL of DMEM containing 10% FBS. The 50 μL of aCD3 and aCD28 (final concentration of 1 μg/mL for each) were added into 96-well plate without or with 50 μL of human PD-L1 protein (final concentation of 10 nM). Then 50 μL of BMS-936559 or PCC0208025 solution with the final concentrations of 0.01, 0.1, 1 and 10 μM were added to the wells and cultured for 72 h. The supernatants were collected for detection of IFN-γ by using human IFN-γ ELISA Kit.
(DOCX)

S3 Table The statitic results and p values for effects of PCC0208025 on IFN-γ level in CD3^+^ cells in vitro. (DOCX)

S4 Table Effects of PCC0208025 on tumor volume in B16-F10 tumor isograft model. B16-F10 tumors mice were administrated by oral gavage with PCC0208025 at 30 mg/kg or 60 mg/kg, twice daily. On days 7, 9, 11, 14, 16, 18 and 20, tumor volumes were detemined.
(DOCX)

S5 Table Effects of PCC0208025 on tumor weight in B16-F10 tumor isograft model. B16-F10 tumors mice were administrated by oral gavage with PCC0208025 at 30 mg/kg or 60 mg/kg, twice daily. On day 20, the tumors were removed and weighed.
(DOCX)

S6 Table Effects of PCC0208025 on plasma IFN-γ level in B16-F10-bearing mice. Before all the mice with tumors were decapitated, the blood samples from orbital venous sinus were collected into tubes with heparin for plasma preparation. Plasma IFN-γ level was determined by using mice ELISA kit.
(DOCX)

S7 Table Effects of PCC0208025 on T cell subsets from tumor in B16-F10-bearing mice. T cell subsets were counted by flow cytometry from tumors in B16-F10-bearing mice. The percentage of CD3^+^, CD3^+^CD8^+^, CD8^+^IFN-γ^+^, CD3^+^CD4^+^, and CD4^+^CD25^+^CD127^low/-^ T cells were presented.
(DOCX)

S8 Table Pharmacokinetics of PCC0208025 in plasma and tumors in melanoma-bearing mice. After a single dose of 60 mg/kg PCC0208025, PCC0208025 concentrations were detected in plasma and tumors at 1h, 3h and 8h, respectively.
(DOCX)

## References

[1] Crunkhorn S. Immunotherapy: Vaccine patch to treat melanoma. *Nat Rev Drug Discov* 2017; 17:18. 10.1038/nrd.2017.259 29282370

[2] Hu Z, Ye L, Xing Y, Hu J, Xi T. Combined SEP and anti-PD-L1 antibody produces a synergistic antitumor effect in B16-F10 melanoma-bearing mice. *Sci Rep* 2018, 8: 217. 10.1038/s41598-017-18641-y 29317734 PMC5760644

[3] Scott LJ. Nivolumab: A Review in Advanced Melanoma. *Drugs* 2015; 75:1413–1424. 10.1007/s40265-015-0442-6 26220912

[4] Lee L, Gupta M, Sahasranaman S. Immune Checkpoint inhibitors: An introduction to the next-generation cancer immunotherapy. *J Clin Pharmacol* 2016; 56:157–169. 10.1002/jcph.591 26183909

[5] Zarganes-Tzitzikas T, Konstantinidou M, Gao Y, Krzemien D, Zak K, Dubin G, et al. Inhibitors of programmed cell death 1 (PD-1): a patent review (2010–2015). *Expert Opin Ther Pat* 2016; 26:973–977. 10.1080/13543776.2016.1206527 27367741 PMC5379636

[6] Zhu Z, Liu W, Gotlieb V. The rapidly evolving therapies for advanced melanoma—Towards immunotherapy, molecular targeted therapy, and beyond. *Crit Rev Oncol Hematol* 2016; 99:91–99. 10.1016/j.critrevonc.2015.12.002 26708040

[7] Yamada H, Hida N, Satoh H, Yamagishi T, Hiroshima Y, Yoshii S, et al. Improved outcomes with pembrolizumab treatment in two cases of double cancer including non-small-cell lung cancer. *Anti-cancer drugs* 2018; 30:105–109. 10.1097/CAD.0000000000000677 30074503

[8] Mahoney KM, Freeman GJ, McDermott DF. The Next Immune-Checkpoint Inhibitors: PD-1/PD-L1 Blockade in Melanoma. *Clin Ther* 2015; 37:764–782. 10.1016/j.clinthera.2015.02.018 25823918 PMC4497957

[9] Magiera-Mularz K, Skalniak L, Zak KM, Musielak B, Rudzinska-Szostak E, Berlicki L, et al. Bioactive Macrocyclic Inhibitors of the PD-1/PD-L1 Immune Checkpoint. *Angew Chem Int Ed Engl* 2017; 56:13732–13735. 10.1002/anie.201707707 28881104 PMC6400216

[10] Sasikumar PG, Ramachandra M. Small-Molecule Immune Checkpoint Inhibitors Targeting PD-1/PD-L1 and Other Emerging Checkpoint Pathways. *BioDrugs* 2018;32: 481–497. 10.1007/s40259-018-0303-4 30168070

[11] Li K, Tian H. Development of small-molecule immune checkpoint inhibitors of PD-1/PD-L1 as a new therapeutic strategy for tumour immunotherapy. *J Drug Target* 2019; 3:244–256. 10.1080/1061186X.2018.1440400 29448849

[12] Skalniak L, Zak KM, Guzik K, Magiera K, Musielak B, Pachota M, et al. Small-molecule inhibitors of PD-1/PD-L1 immune checkpoint alleviate the PD-L1-induced exhaustion of T-cells. *Oncotarget* 2017; 8:72167–72181. 10.18632/oncotarget.20050 29069777 PMC5641120

[13] Zak KM, Grudnik P, Guzik K, Zieba BJ, Musielak B, Domling A, et al. Structural basis for small molecule targeting of the programmed death ligand 1 (PD-L1). *Oncotarget* 2016; 7:30323–30335. 10.18632/oncotarget.8730 27083005 PMC5058683

[14] Udhwani T, Mukherjee S, Sharma K, Sweta J, Khandekar N, et al. Design of PD-L1 inhibitors for lung cancer. *Bioinformation* 2019, 28: 139–150. 10.6026/97320630015139 PMC6677907 31435160

[15] Weinmann H. Cancer Immunotherapy: Selected Targets and Small-Molecule Modulators. *ChemMedChem* 2016; 11:450–466. 10.1002/cmdc.201500566 26836578

[16] Chupak LS, Zheng X. Compounds useful as immunomodulators. Bristol-Myers Squibb Company. 2015, WO2015034820 A1.

[17] Lv G, Sun D, Zhang J, Xie X, Wu X, Fang W, et al. Lx2-32c, a novel semi-synthetic taxane, exerts antitumor activity against prostate cancer cells in vitro and in vivo. *Acta Pharm Sin B* 2017; 7:52–58. 10.1016/j.apsb.2016.06.005 28119808 PMC5237719

[18] Yang Y, Guan D, Lei L, Lu J, Liu JQ, Yang G, et al. H6, a novel hederagenin derivative, reverses multidrug resistance in vitro and in vivo. *Toxicol Appl Pharmacol* 2018; 341:98–105. 10.1016/j.taap.2018.01.015 29408042

[19] Ma YT, Yang Y, Cai P, Sun DY, Sanchez-Murcia PA, Zhang XY, et al. A Series of Enthalpically Optimized Docetaxel Analogues Exhibiting Enhanced Antitumor Activity and Water Solubility. *J Nat Prod* 2018; 81:524–533. 10.1021/acs.jnatprod.7b00857 29359935

[20] Zhang D, Xu Q, Wang N, Yang Y, Liu J, Yu G, et al. A complex micellar system co-delivering curcumin with doxorubicin against cardiotoxicity and tumor growth. *Int J Nanomedicine* 2018; 13:4549–4561. 10.2147/IJN.S170067 30127606 PMC6091483

[21] Meng X, Du G, Ye L, Sun S, Liu Q, Wang H, et al. Combinatorial antitumor effects of indoleamine 2,3-dioxygenase inhibitor NLG919 and paclitaxel in a murine B16-F10 melanoma model. *Int J Immunopath Ph* 2017; 30:215–226. 10.1177/0394632017714696 PMC5815254 28604143

[22] Sun S, Du G, Xue J, Ma J, Ge M, Wang H, et al. PCC0208009 enhances the anti-tumor effects of temozolomide through direct inhibition and transcriptional regulation of indoleamine 2,3-dioxygenase in glioma models. *Int J Immunopathol Pharmacol* 2018; 32:2058738418787991. 10.1177/2058738418787991 PMC6047256 29993291

[23] Toor SM, Syed Khaja AS, Alkurd I, Elkord E. In-vitro effect of pembrolizumab on different T regulatory cell subsets. *Clin Exp Immunol* 2018; 191:189–197. 10.1111/cei.13060 28963773 PMC5758372

[24] Kim HM, Lim J, Yoon YD, Ahn JM, Kang JS, Lee K, et al. Anti-tumor activity of ex vivo expanded cytokine-induced killer cells against human hepatocellular carcinoma. *Int Immunopharmacol* 2007; 7:1793–1801. 10.1016/j.intimp.2007.08.007 17996690

[25] Whiteside TL. FOXP3+ Treg as a therapeutic target for promoting anti-tumor immunity. *Expert Opin Ther Targets* 2018; 22: 10.1080/14728222.2018.1451514 29532697 PMC6126897

[26] Islas-Vazquez L, Prado-Garcia H, Aguilar-Cazares D, Meneses-Flores M, Galicia-Velasco M, Romero-Garcia S, et al. LAP TGF-Beta Subset of CD4(+)CD25(+)CD127(-) Treg Cells is Increased and Overexpresses LAP TGF-Beta in Lung Adenocarcinoma Patients. *Biomed Res Int* 2015; 2015:430943. 10.1155/2015/430943 PMC4637030 26582240

[27] Wang W, Lau R, Yu D, Zhu W, Korman A, Weber J. PD1 blockade reverses the suppression of melanoma antigen-specific CTL by CD4+ CD25(Hi) regulatory T cells. *Int Immunol* 2009; 21:1065–1077. 10.1093/intimm/dxp072 19651643 PMC2731790

