# Correction: PCC0208025 (BMS202), a small molecule inhibitor of PD-L1, produces an antitumor effect in B16-F10 melanoma-bearing mice

**DOI:** 10.1371/journal.pone.0251020

**Published:** 2021-04-28

**Authors:** The PLOS ONE Staff

## Notice of republication

This article was republished on April 8, 2021, to correct errors in the Data Availability Statement that were introduced during the typesetting process. The publisher apologizes for the errors. Please download this article again to view the correct version. The originally published, uncorrected article and the republished, corrected articles are provided here for reference.

## Supporting information

S1 File Originally published, uncorrected article. (PDF)

S2 File Republished, corrected article. (PDF)
