# Supplementary material for: PCC0208025 (BMS202), a small molecule inhibitor of PD-L1, produces an antitumor effect in B16-F10 melanoma-bearing mice
Source: PLoS One. 2020 Mar 26;15(3):e0228339. doi: 10.1371/journal.pone.0228339 (PMC7098565; doi:10.1371/journal.pone.0228339)
Supplement: S1 Fig — (PDF) [file pone.0228339.s001.PDF]

File D:\DATA\ Tgt Mass (EZX):

Injection Date : 23 Jun 16 2:03 pm +0800 Seq. Line : 0
Sample Name : Location :
Acq. Operator : Inj : 1
Spec. Reported : MS Integration Inj Volume : 1 ul
Acq. Method : D:\METHODS\2-POS-MON-1.M
Analysis Method : D:\METHODS\2-POS-MON-1.M
Sample Info : Easy-Access Method: '1-POS-MON-1'
Method Info : Column:Xbridge C18(2) (4.6x 50 mm, 3.5um)
Mobile phase: H2O(10 mmol NH4HCO3) (A) / ACN(B)
Elution program: Gradient from 10 to 95% of B in1.5min at 1.8ml/min
Temperature: 50 °C
Detection: UV (214 , 4 nm) and MS (ESI, Pos mode ,110 to 1100 amu)

*DAD1 A, Sig=214,4 Ref=off

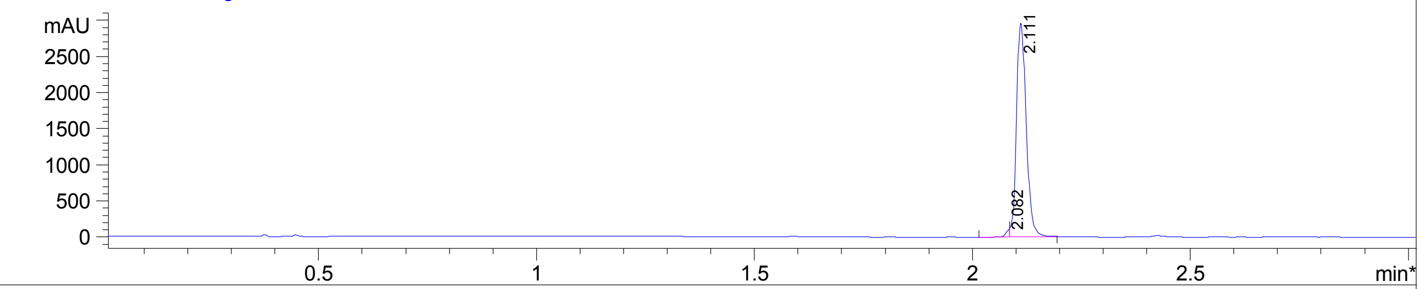

*DAD1 B, Sig=254,4 Ref=off

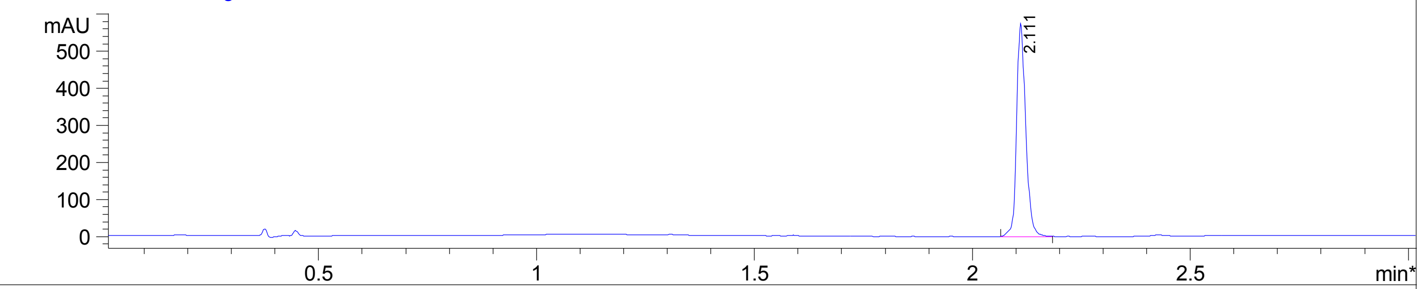

MSD1 TIC, MS File ES-API, Pos, Scan, Frag: 80

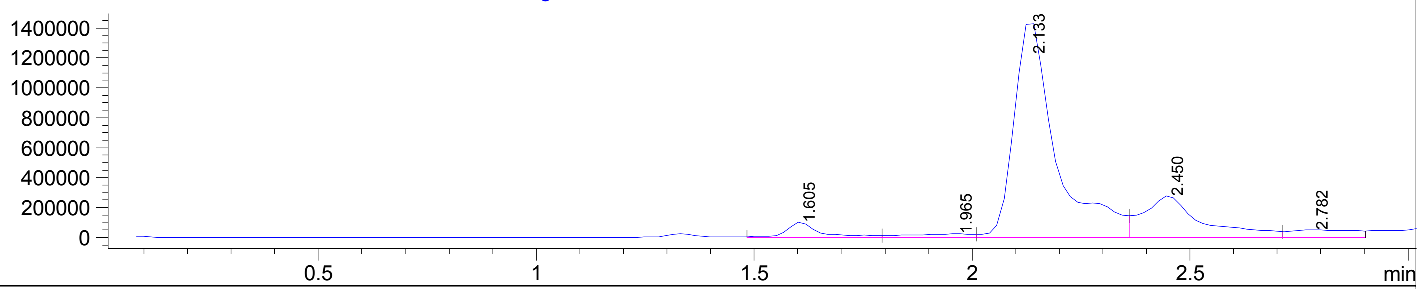

Integration Results for DAD1 A, Sig=214,4 Ref=off

RetTim	Width	Area	Height	Area%
2.08	0.01	64.27	96.93	1.38
2.11	0.02	4606.39	2962.74	98.62

Integration Results for DAD1 B, Sig=254,4 Ref=off

RetTim	Width	Area	Height	Area%
2.11	0.02	836.38	574.62	100.00

Ret. Time: 1.61 <<<< POSITIVE SPECTRA >>>>

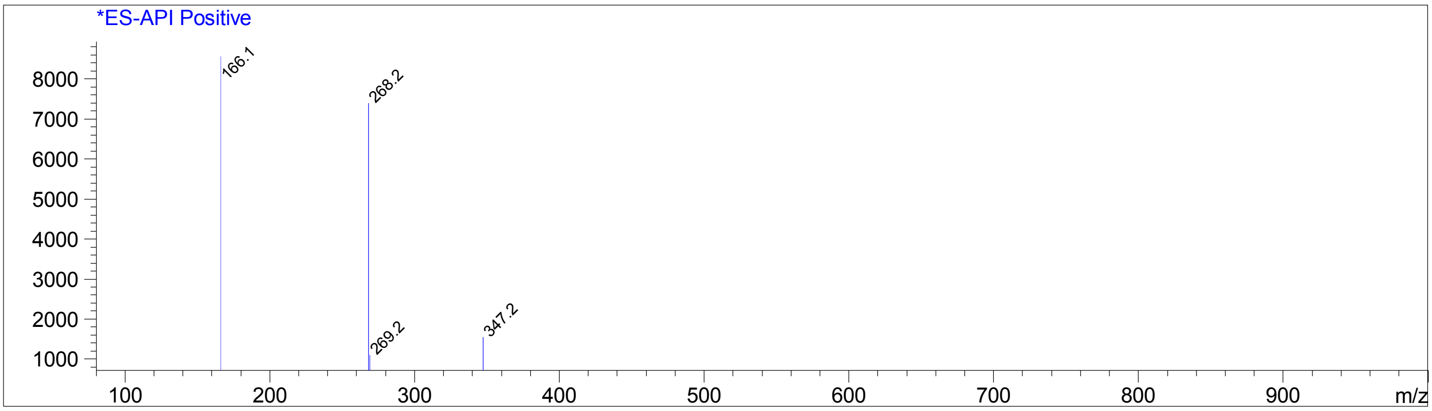

Ret. Time: 1.97

*ES-API Positive <<< Below Cutoff >>>

Ret. Time: 2.13

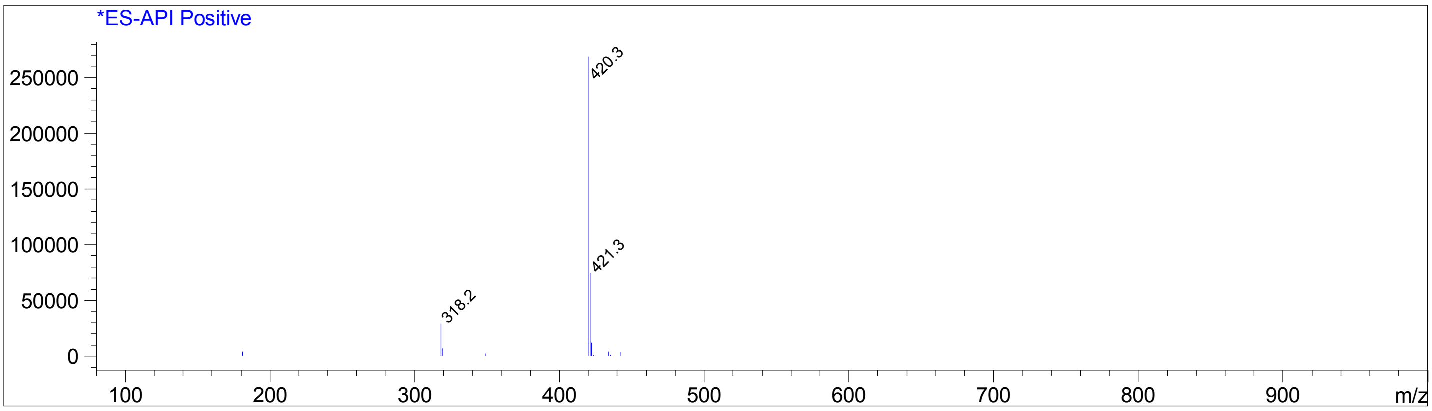

Ret. Time: 2.45

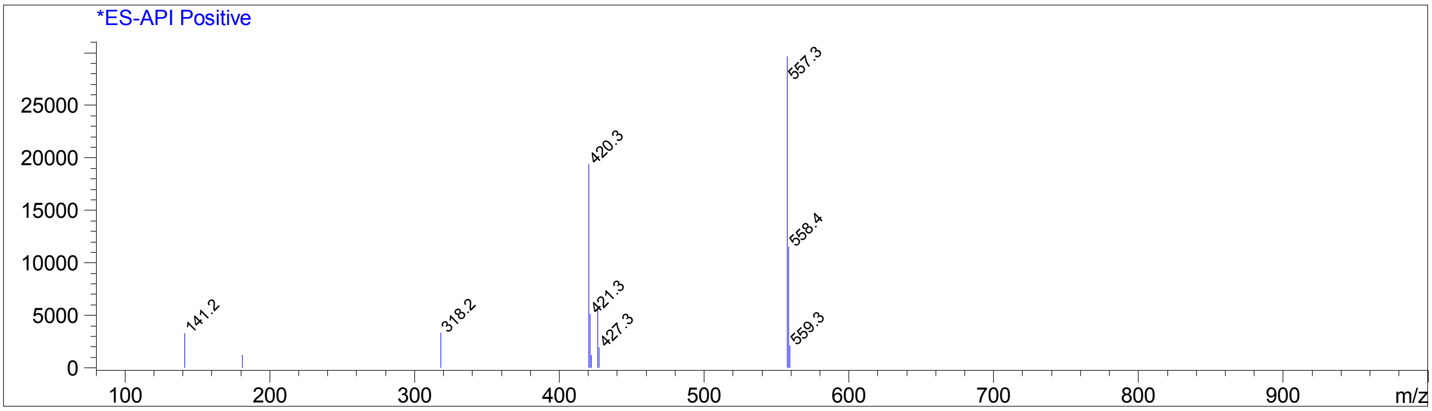

Ret. Time: 2.78

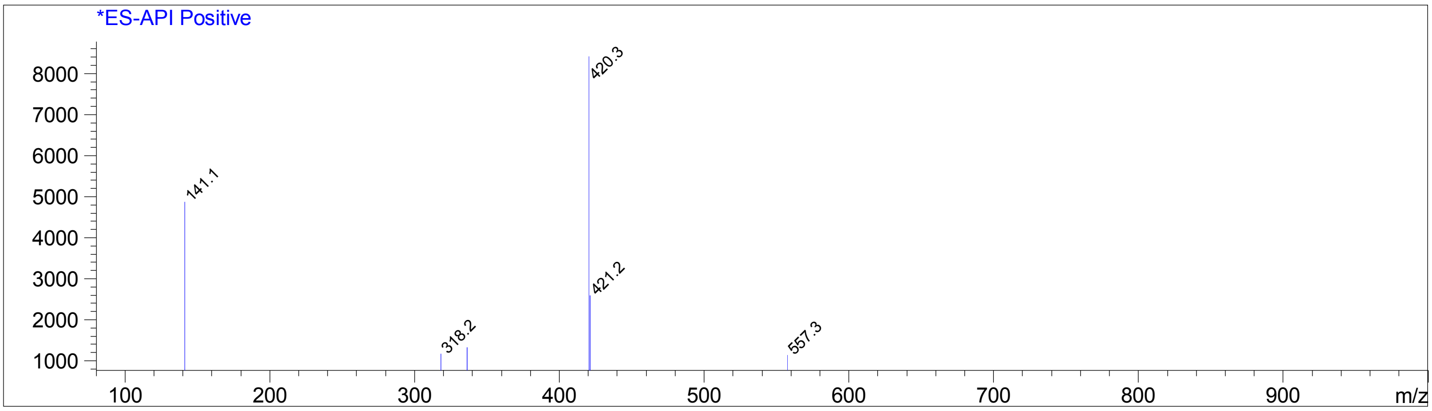
*ES-API Positive
8000
7000
6000
5000
4000
3000
2000
1000
141.1
318.2
420.3
421.2
557.3
100
200
300
400
500
600
700
800
900
m/z
