# Supplementary figures and images for: PCC0208025 (BMS202), a small molecule inhibitor of PD-L1, produces an antitumor effect in B16-F10 melanoma-bearing mice
Source: PLoS One. 2020 Mar 26;15(3):e0228339. doi: 10.1371/journal.pone.0228339 (PMC7098565; doi:10.1371/journal.pone.0228339)

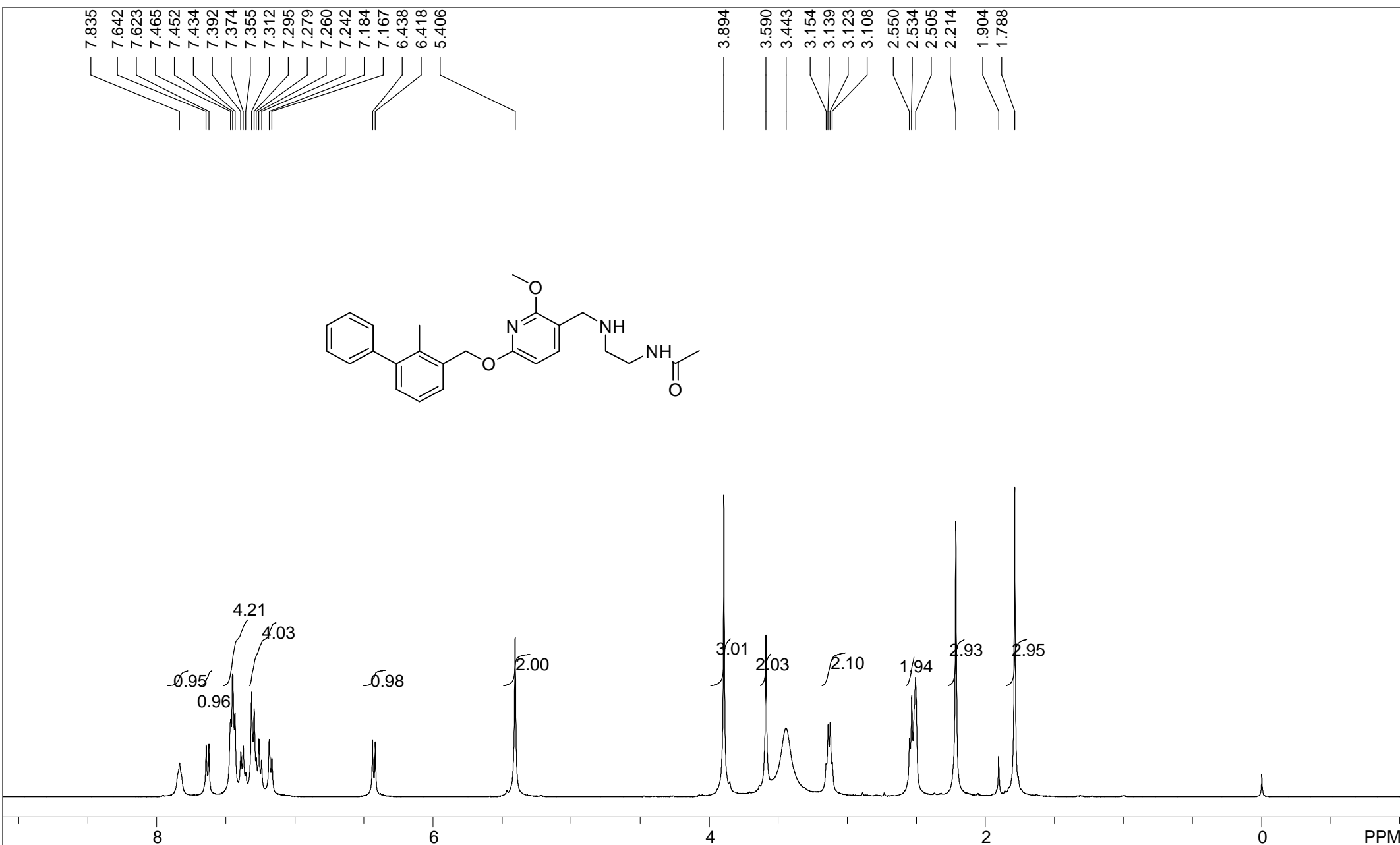

|           |           |               |             |                                            |              |                |  |
|-----------|-----------|---------------|-------------|--------------------------------------------|--------------|----------------|--|
| nmr DMSO, |           |               |             | USER: nmr-- DATE: Thu Jun 23 08:21:35 2016 |              |                |  |
| F1: 400.  | F2: 1.000 | SW1: 8224     |             | OF1: 2468.6                                | PTS1d: 32768 |                |  |
| EX: zg30  |           | PW: 13.0 usec | PD: 1.0 sec | NA: 8                                      | LB: 0.0      | Nuts - \$pdata |  |

Supplement: S2 Fig — (PDF) [file pone.0228339.s002.pdf]
