# Supplementary material for: PCC0208025 (BMS202), a small molecule inhibitor of PD-L1, produces an antitumor effect in B16-F10 melanoma-bearing mice
Source: PLoS One. 2020 Mar 26;15(3):e0228339. doi: 10.1371/journal.pone.0228339 (PMC7098565; doi:10.1371/journal.pone.0228339)

CD3^{+}, Control group

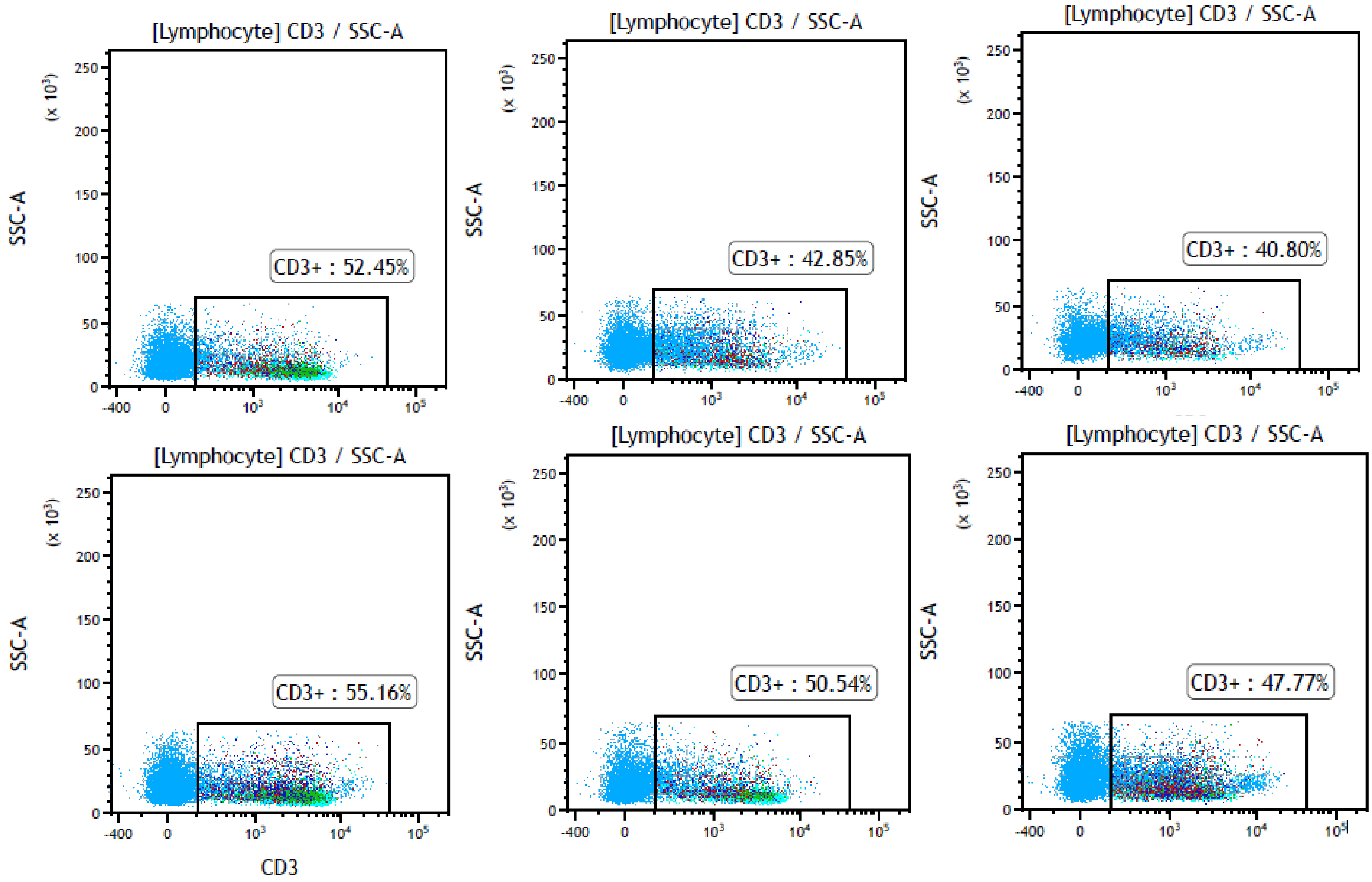

CD3^{+}, 30 mg/kg group

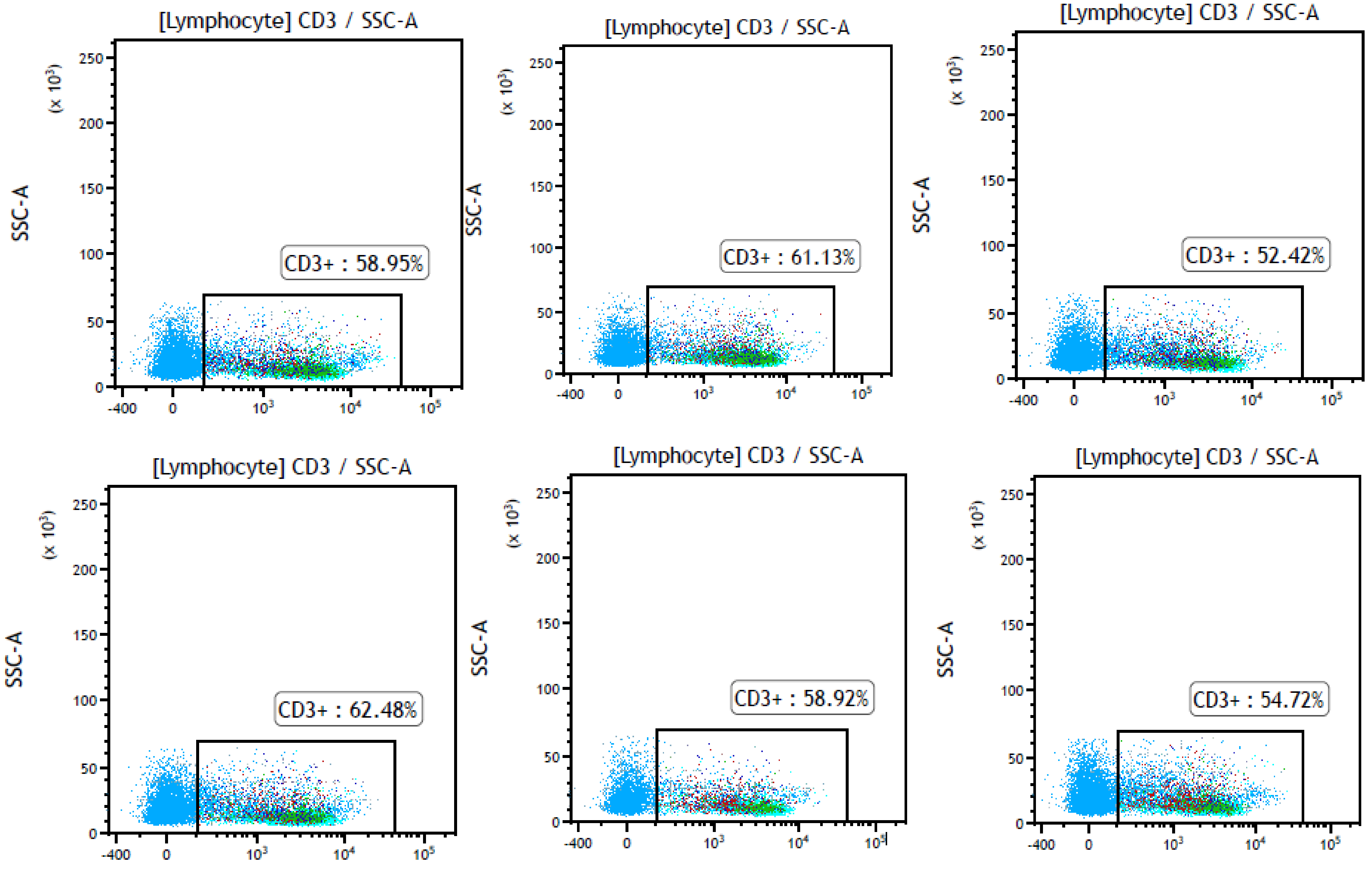

CD3^{+}, 60 mg/kg group

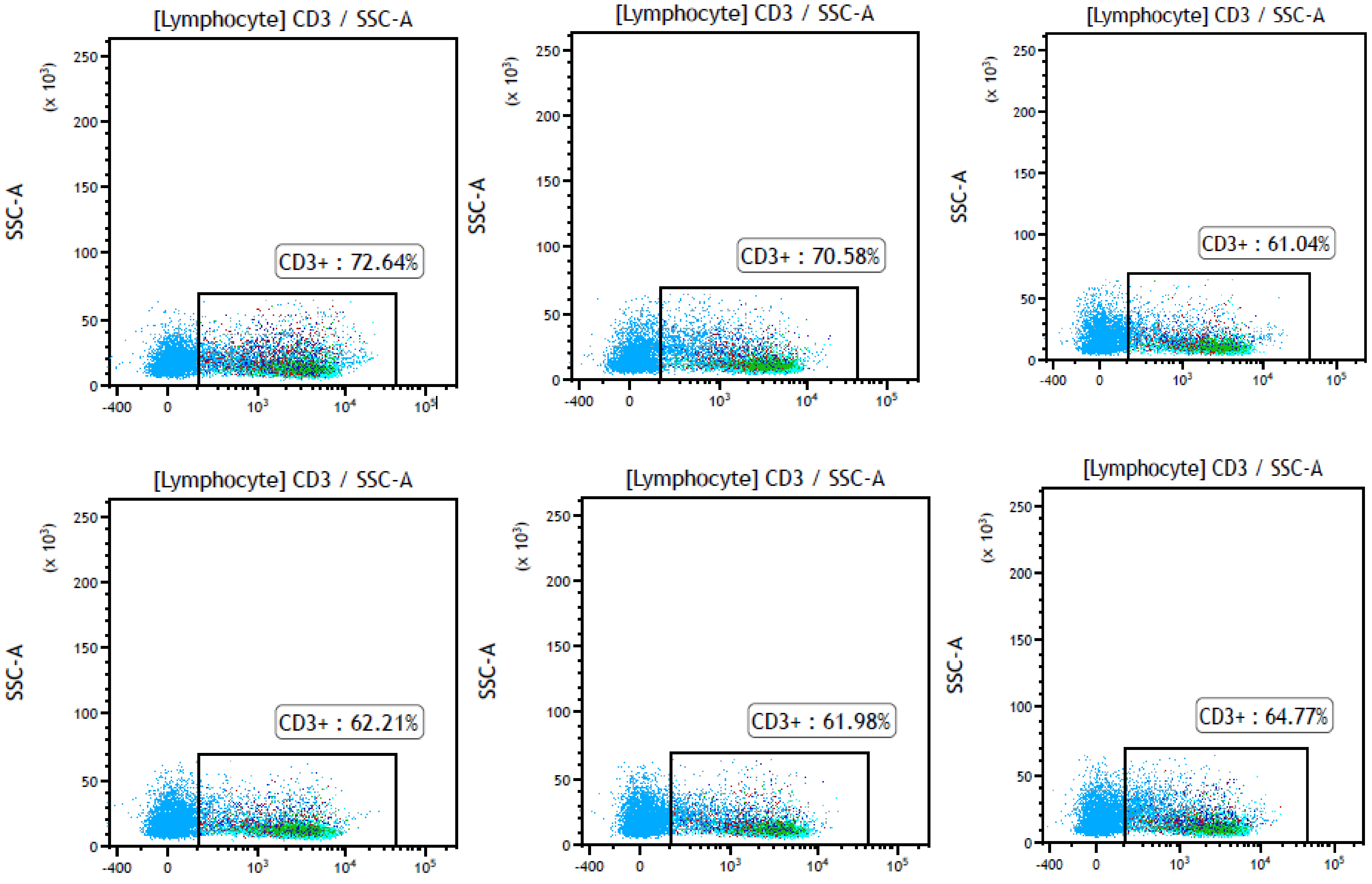

$CD4^{+}CD8^{+}$, Control group

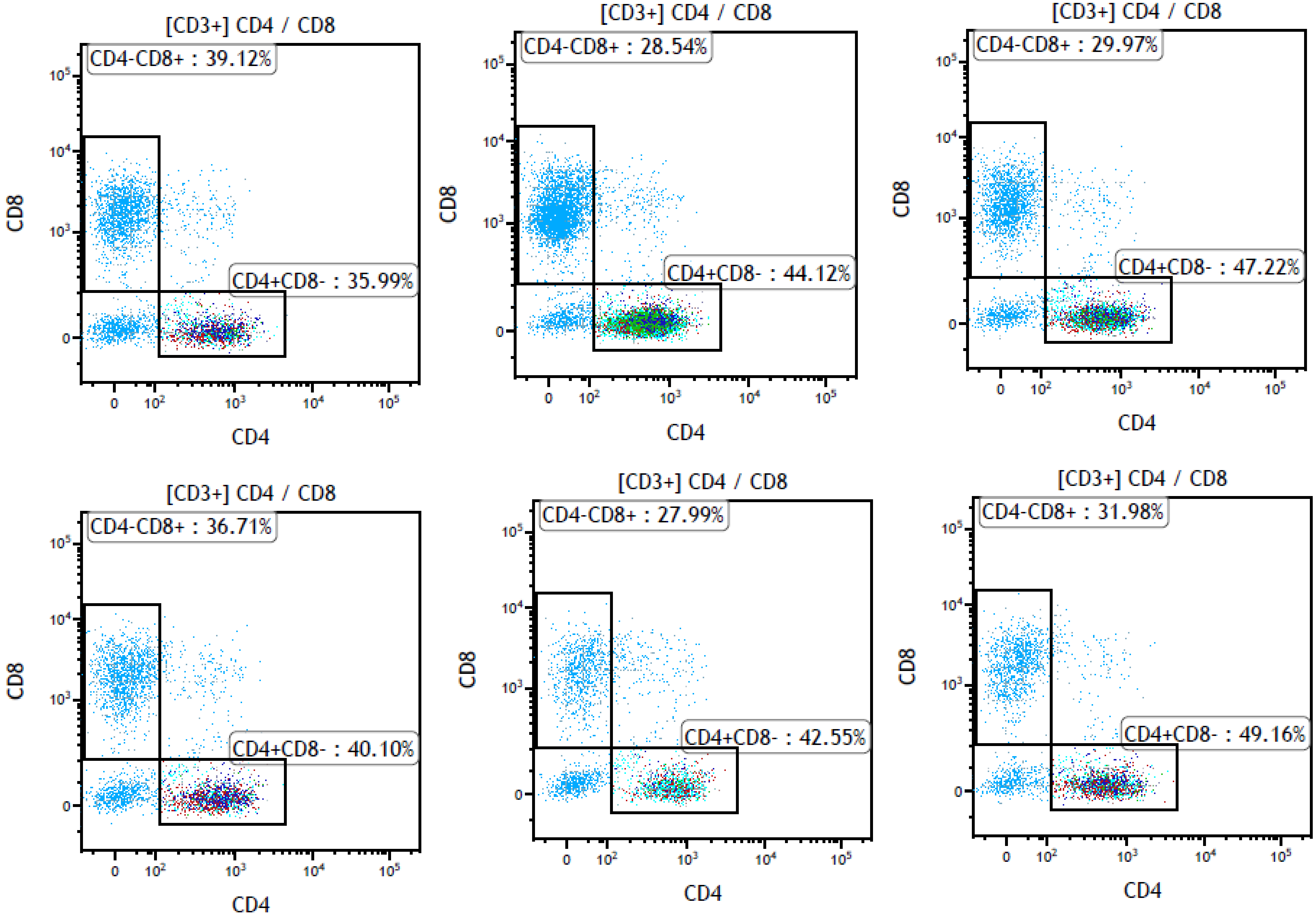

CD4$^+$CD8$^+$, 30 mg/kg group

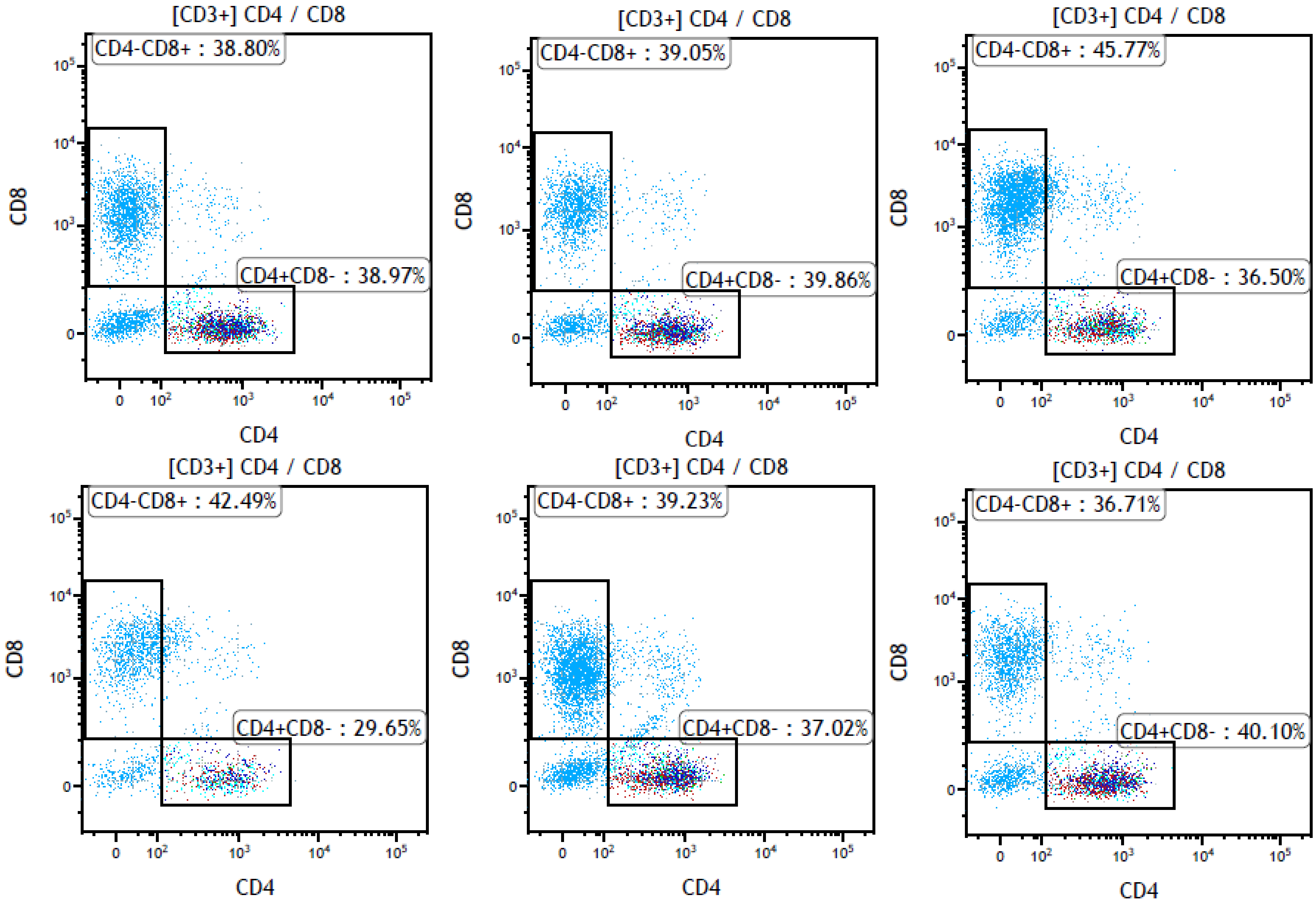

CD4^{+}CD8^{+}, 60 mg/kg group

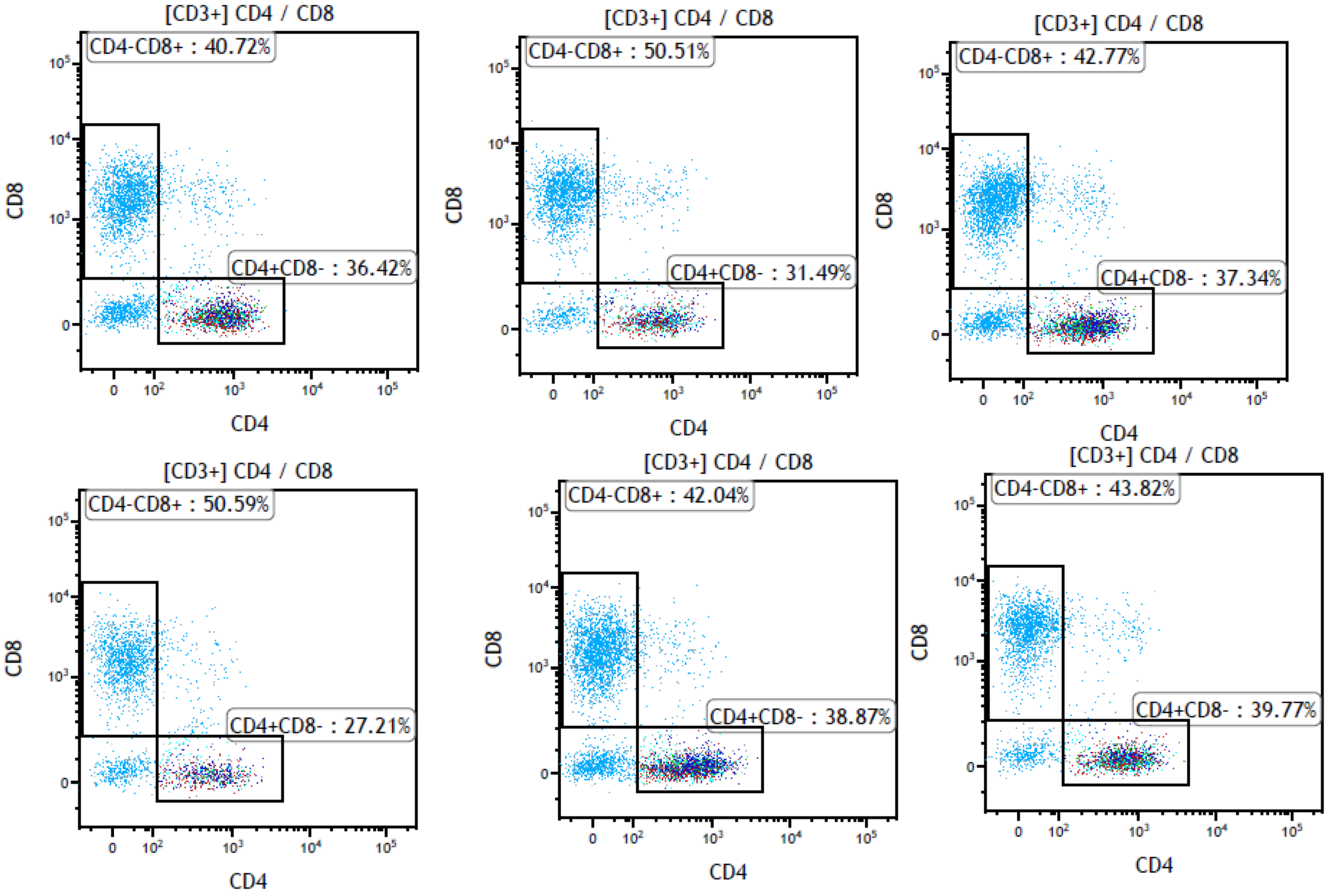

Treg, Control group

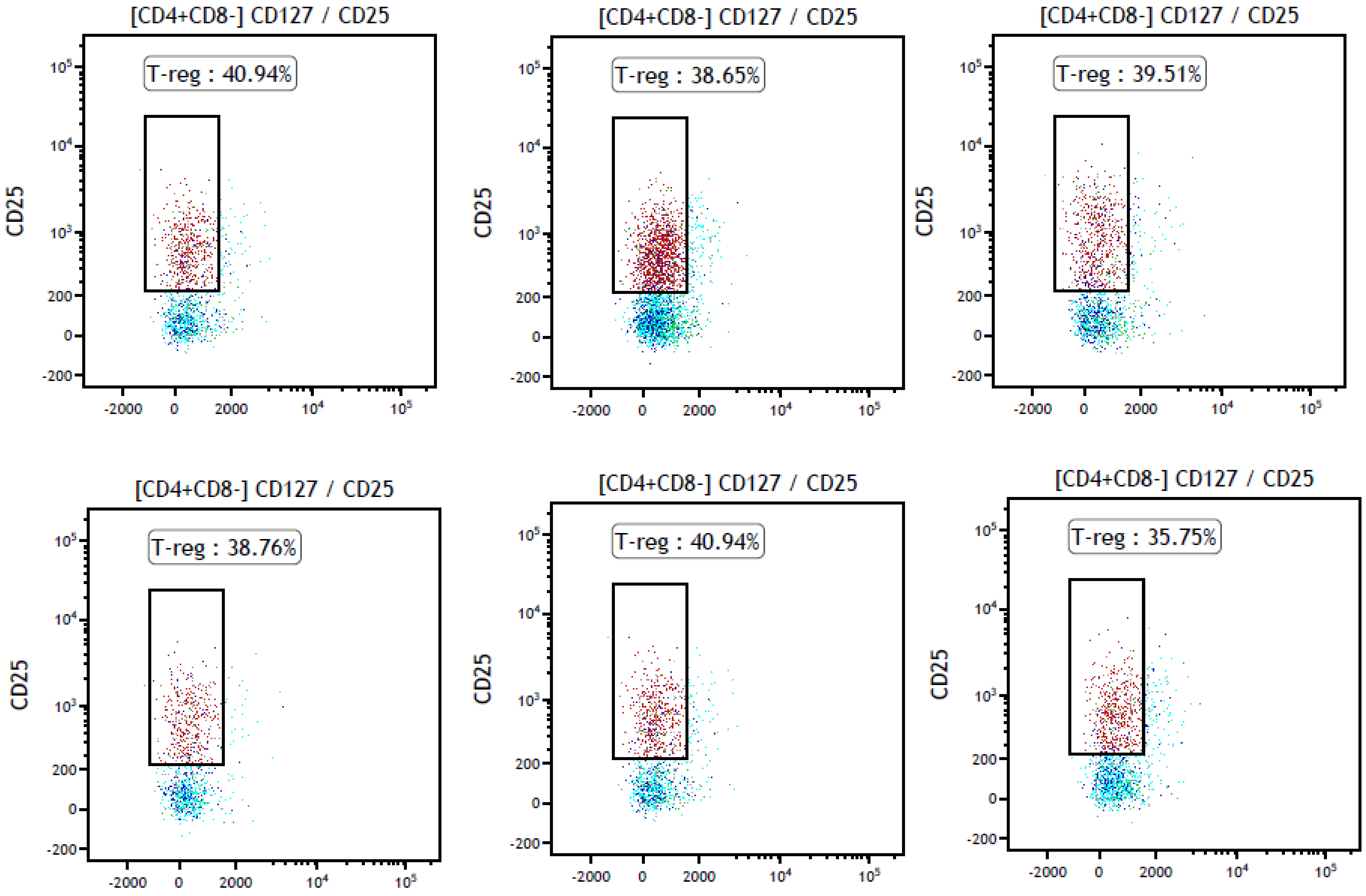

Treg, 30 mg/kg group

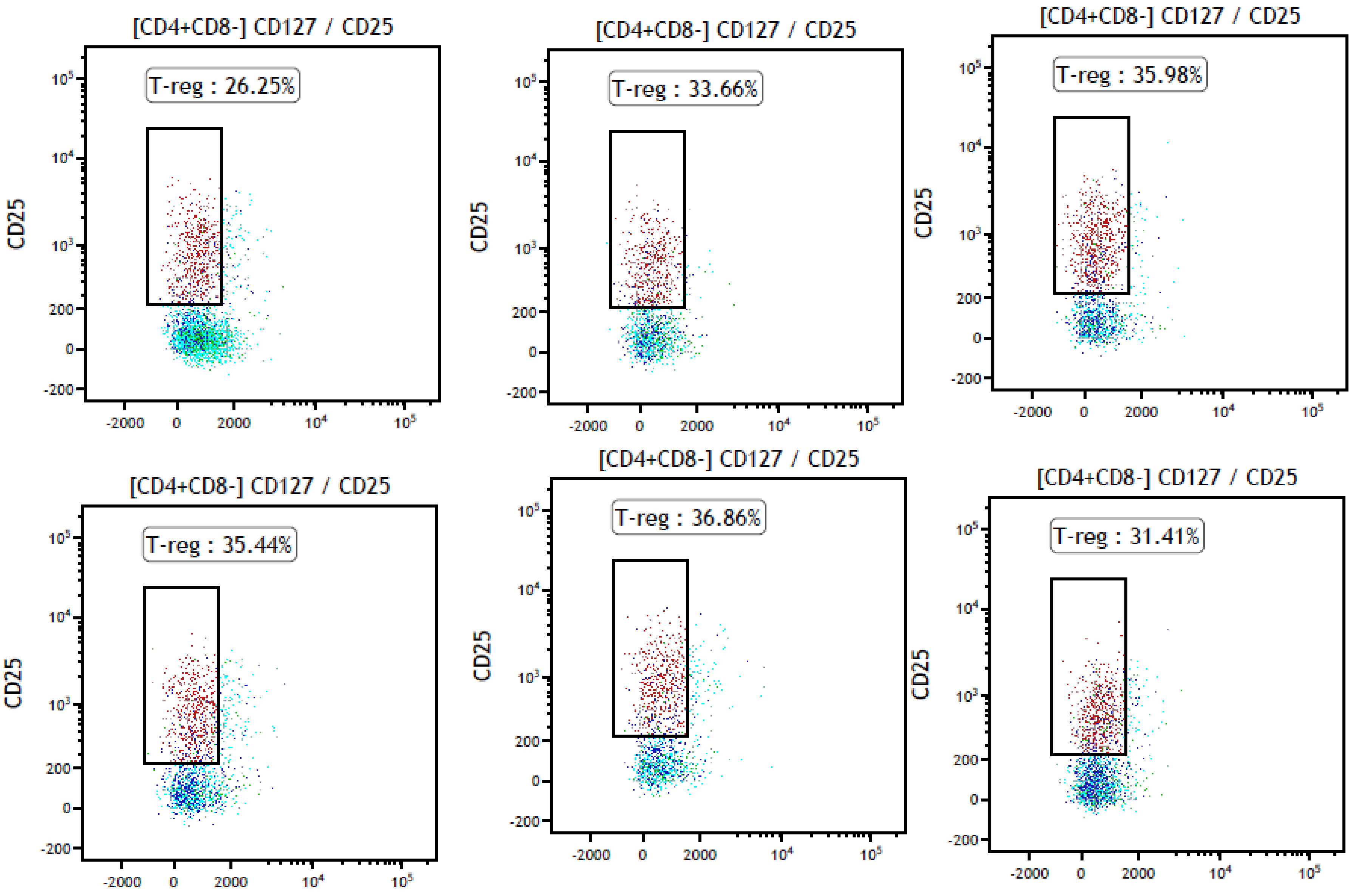

Treg, 60 mg/kg group

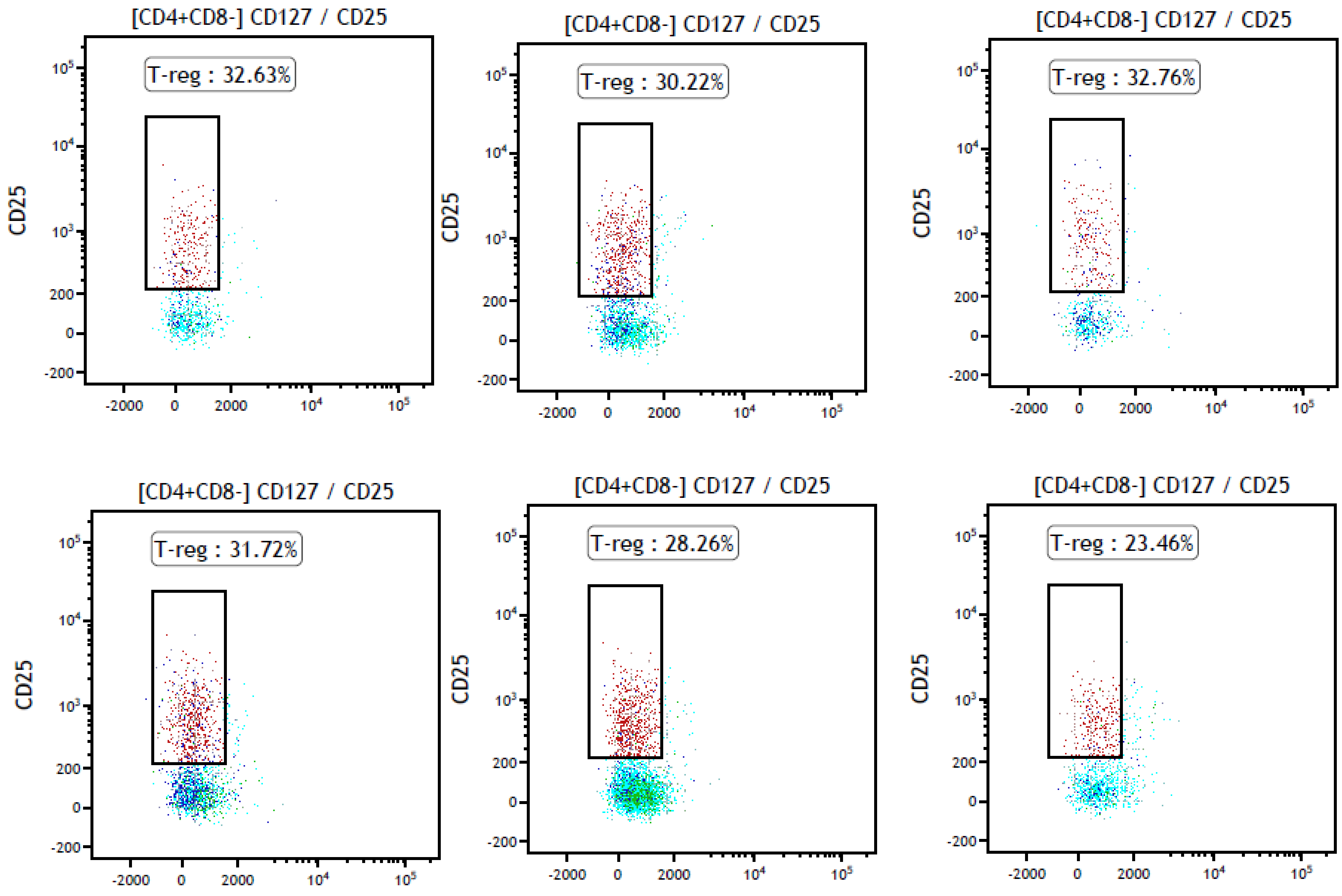

CD8$^+$IFN-gamma, Control group

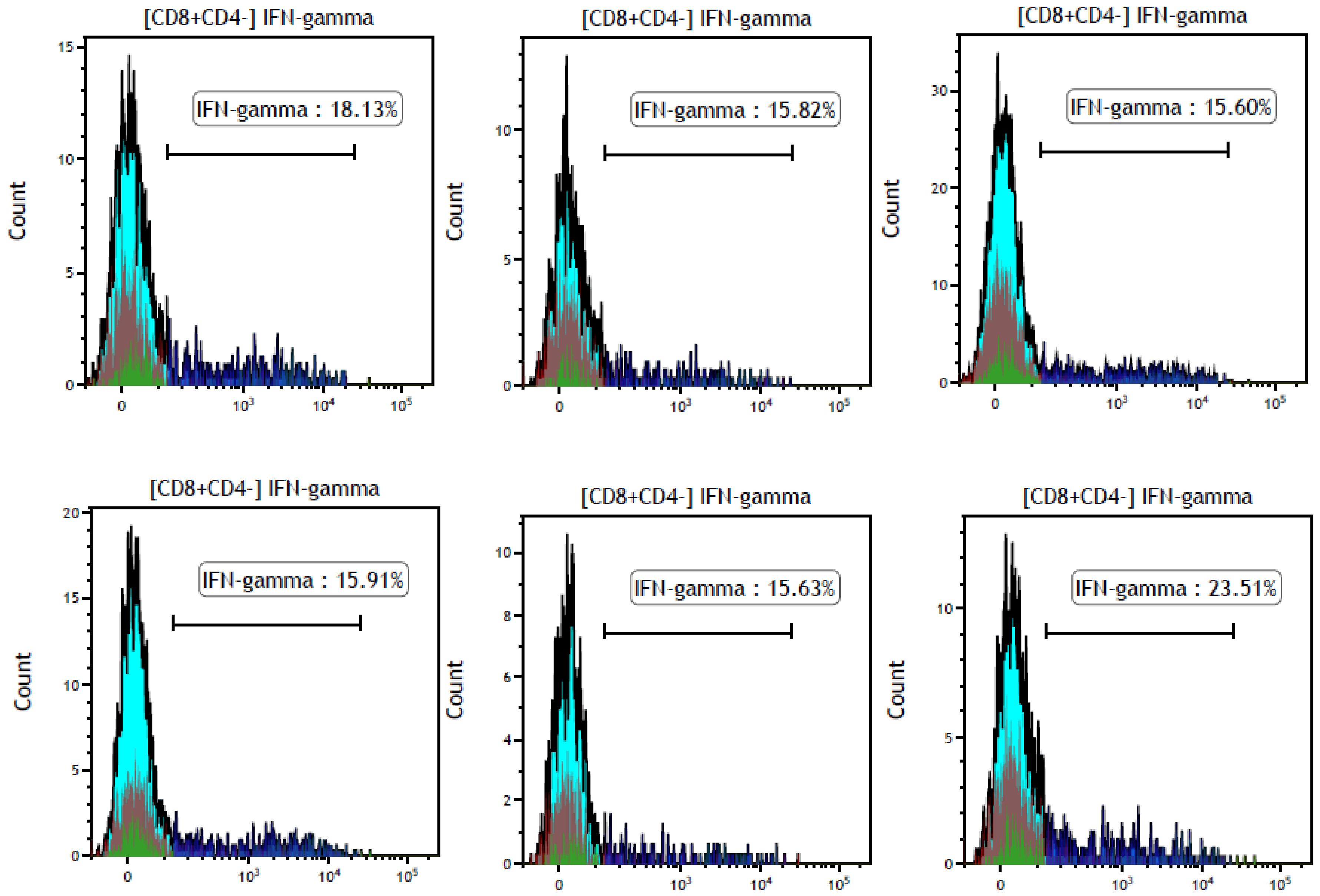

CD8$^+$IFN-gamma, 30 mg/kg group

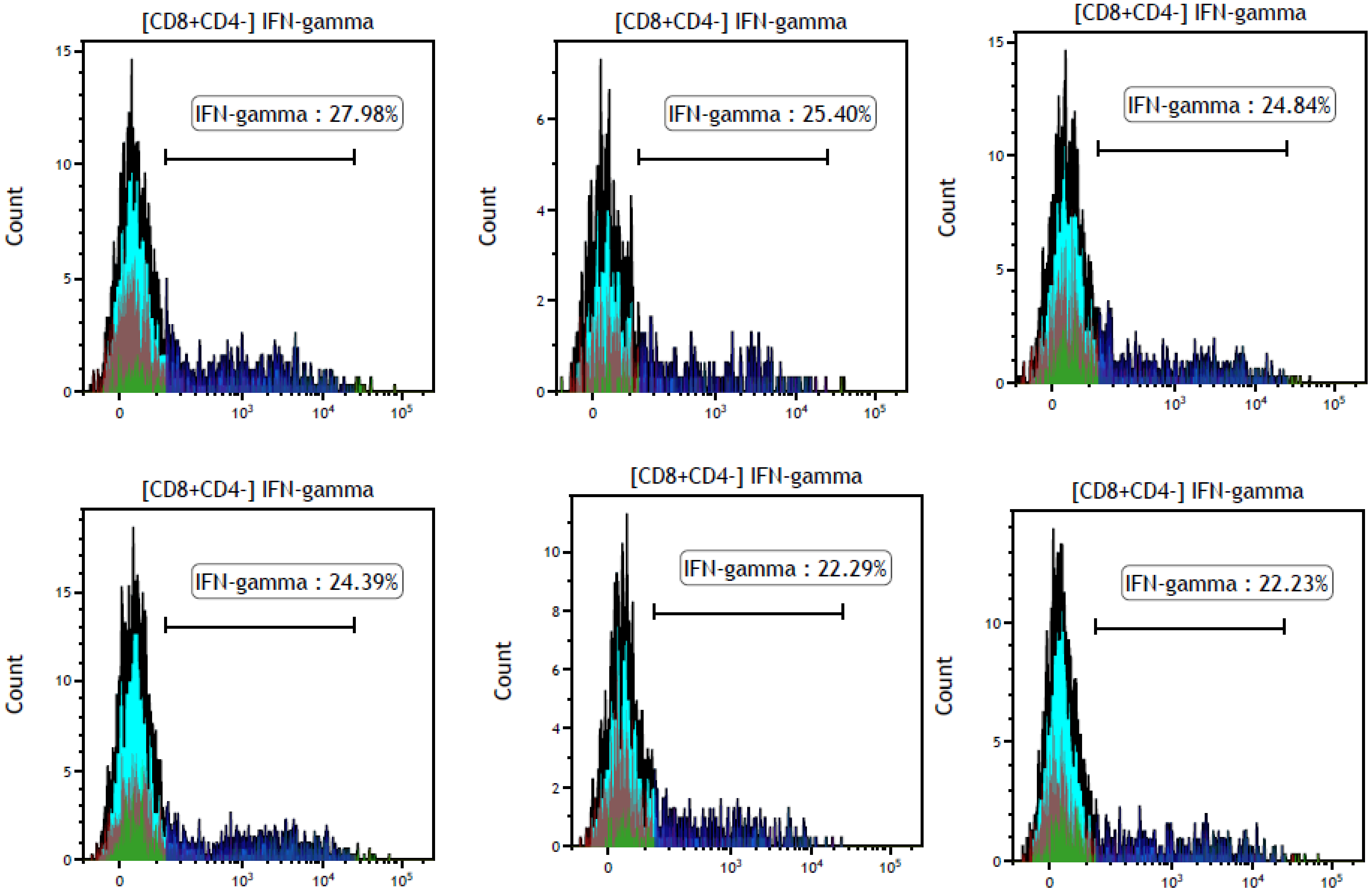

CD8$^+$IFN-gamma, 60 mg/kg group

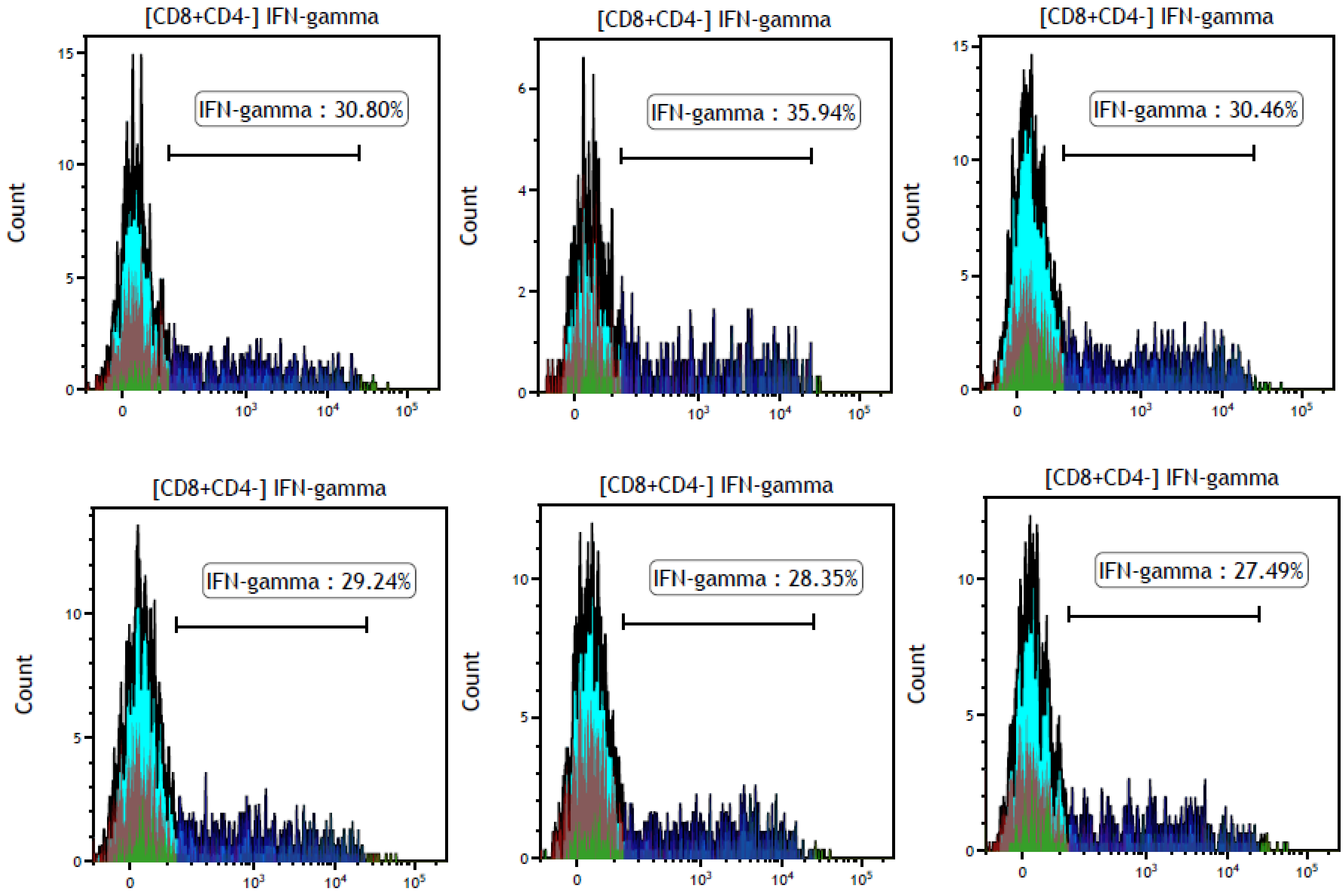

Supplement: S3 Fig — (PDF) [file pone.0228339.s003.pdf]
