# Supplementary material for: PCC0208025 (BMS202), a small molecule inhibitor of PD-L1, produces an antitumor effect in B16-F10 melanoma-bearing mice
Source: PLoS One. 2020 Mar 26;15(3):e0228339. doi: 10.1371/journal.pone.0228339 (PMC7098565; doi:10.1371/journal.pone.0228339)
Supplement: S1 Table — In HTRF assay, Tag2-PD-1 (20 nM final), PCC0208025 (0.15, 0.46, 1.37, 4.12, 12.35, 37.04, 111.11, 333.33 and 1000 nM, final) or BMS-936559 (0.002, 0.006, 0.024, 0.10, 0.39, 1.56, 6.25, 25 and 100 nM final), and Tag1-PD-L1 (2 nM final) were designed. After pre-incubation, anti-Tag1-EuK and anti-Tag2-XL665 was added into the assay well. The signals (665 nm/620 nm ratio) were obtained on Tecan M200 PRO. HTRF ratio = (OD665 nm/OD620 nm) × 104. (DOCX) [file pone.0228339.s004.docx]

| BMS-936559 | | |
| --- | --- | --- |
| Log concentration (nM) | HTRF ratio Sample 1 | HTRF ratio Sample 2 |
| 2.000 | 543 | 450 |
| 1.39794 | 577 | 446 |
| 0.79588 | 539 | 689 |
| 0.1931246 | 559 | 645 |
| -0.4089354 | 638 | 568 |
| -1.000 | 1484 | 1658 |
| -1.619789 | 1737 | 1932 |
| -2.221849 | 1905 | 1867 |
| -2.69897 | 1897 | 2058 |
| PCC0208025 | | |
| Log concentration (nM) | HTRF ratio Sample 1 | HTRF ratio Sample 2 |
| 3.000 | 579 | 678 |
| 2.522874 | 1175 | 1056 |
| 2.045753 | 1404 | 1278 |
| 1.568671 | 1510 | 1378 |
| 1.091667 | 1697 | 1557 |
| 0.6148972 | 1473 | 1578 |
| 0.1367206 | 1584 | 1500 |
| -0.3372422 | 1595 | 1523 |
| -0.8239087 | 1667 | 1544 |
