# Supplementary material for: PCC0208025 (BMS202), a small molecule inhibitor of PD-L1, produces an antitumor effect in B16-F10 melanoma-bearing mice
Source: PLoS One. 2020 Mar 26;15(3):e0228339. doi: 10.1371/journal.pone.0228339 (PMC7098565; doi:10.1371/journal.pone.0228339)
Supplement: S2 Table — Human CD3+ cells were plated in the 96-well plate with a density of 1 × 10^5^ cells/well in 100 μL of DMEM containing 10% FBS. The 50 μL of aCD3 and aCD28 (final concentration of 1 μg/mL for each) were added into 96-well plate without or with 50 μL of human PD-L1 protein (final concentation of 10 nM). Then 50 μL of BMS-936559 or PCC0208025 solution with the final concentrations of 0.01, 0.1, 1 and 10 μM were added to the wells and cultured for 72 h. The supernatants were collected for detection of IFN-γ by using human IFN-γ ELISA Kit. (DOCX) [file pone.0228339.s005.docx]

| Groups | IFN-γl (pg/ml) | | | | | | | | | | |
| --- | --- | --- | --- | --- | --- | --- | --- | --- | --- | --- | --- |
| aCD3/aCD28 (1 μg/mL) | - | + | + | + | + | + | + | + | + | + | + |
| PD-L1 protein (10 nM) | - | - | + | + | + | + | + | + | + | + | + |
| PCC0208025 (mM) | - | - | - | 0.01 | 0.1 | 1 | 10 | - | - | - | - |
| BMS-936559 (mM) | - | - | - | - | - | - | - | 0.01 | 0.1 | 1 | 10 |
| Sample 1 | 23.1 | 1256.0 | 145.3 | 706.1 | 945.6 | 723.5 | 435.3 | 656.5 | 903.3 | 689.3 | 713.3 |
| Sample 2 | 47.2 | 1034.1 | 245.6 | 658.5 | 689.7 | 908.8 | 587.5 | 739.3 | 718.5 | 1087.1 | 879.6 |
| Sample 3 | 34.1 | 1324.9 | 178.5 | 523.4 | 789.4 | 857.7 | 523.4 | 678.5 | 734.6 | 879.7 | 897.4 |
| Sample 4 | 16.6 | 989.3 | 239.9 | 8167 | 768.3 | 798.3 | 468.7 | 756.3 | 849.3 | 934.3 | 945.5 |
| Sample 5 | 37.5 | 1324.5 | 287.8 | 690.5 | 1056.6 | 824.3 | 398.5 | 638.1 | 876.1 | 828.6 | 779.6 |
| Sample 6 | 35.0 | 834.4 | 118.1 | 650.6 | 850.5 | 997.0 | 576.3 | 687.8 | 679.8 | 756.5 | 798.4 |
