# Supplementary material for: PCC0208025 (BMS202), a small molecule inhibitor of PD-L1, produces an antitumor effect in B16-F10 melanoma-bearing mice
Source: PLoS One. 2020 Mar 26;15(3):e0228339. doi: 10.1371/journal.pone.0228339 (PMC7098565; doi:10.1371/journal.pone.0228339)
Supplement: S3 Table — (DOCX) [file pone.0228339.s006.docx]

|  | Blank control | aCD3/aCD28 group | aCD3/aCD28 and PD-L1 |
| --- | --- | --- | --- |
| Blank control | — | — | — |
| aCD3/aCD28 | *P* < 0.001 | — | — |
| aCD3/aCD28 and PD-L1 | *P* = 0.008 | *P* < 0.001 | — |
| PCC0208025 | *P* < 0.001 | *P* < 0.001 | *P* < 0.001 |
| PCC0208025 | *P* < 0.001 | *P* < 0.001 | *P* < 0.001 |
| PCC0208025 | *P* < 0.001 | *P* < 0.001 | *P* < 0.001 |
| PCC0208025 | *P* < 0.001 | *P* < 0.001 | *P* < 0.001 |
| BMS-936559 | *P* < 0.001 | *P* < 0.001 | *P* < 0.001 |
| BMS-936559 | *P* < 0.001 | *P* < 0.001 | *P* < 0.001 |
| BMS-936559 | *P* < 0.001 | *P* < 0.001 | *P* < 0.001 |
| BMS-936559 | *P* < 0.001 | *P* < 0.001 | *P* < 0.001 |
