# Supplementary material for: PCC0208025 (BMS202), a small molecule inhibitor of PD-L1, produces an antitumor effect in B16-F10 melanoma-bearing mice
Source: PLoS One. 2020 Mar 26;15(3):e0228339. doi: 10.1371/journal.pone.0228339 (PMC7098565; doi:10.1371/journal.pone.0228339)
Supplement: S4 Table — B16-F10 tumors mice were administrated by oral gavage with PCC0208025 at 30 mg/kg or 60 mg/kg, twice daily. On days 7, 9, 11, 14, 16, 18 and 20, tumor volumes were detemined. (DOCX) [file pone.0228339.s007.docx]

| Days | Control, Tumor volume (mm^3^) | | | | | | | |
| --- | --- | --- | --- | --- | --- | --- | --- | --- |
| 7 | 96.8 | 78.0 | 116.5 | 105.2 | 115.3 | 109.5 | 89.3 | 95.1 |
| 9 | 117.1 | 128.1 | 308.0 | 200.9 | 142.0 | 175.8 | 180.9 | 112.1 |
| 11 | 233.3 | 191.3 | 545.5 | 290.6 | 204.4 | 304.8 | 261.0 | 130.6 |
| 14 | 305.3 | 661.1 | 1043.8 | 543.9 | 279.9 | 723.7 | 540.5 | 242.7 |
| 16 | 550.8 | 1130.1 | 1887.5 | 842.7 | 289.4 | 881.7 | 823.0 | 291.7 |
| 18 | 821.6 | 2596.3 | 2419.7 | 1022.7 | 539.1 | 1375.0 | 1622.4 | 373.7 |
| 20 | 1678.0 | 3376.5 | 3249.8 | 1515.7 | 1150.0 | 2051.5 | 1813.7 | 1367.0 |

| Days | PCC0208025 30 mg/kg, Tumor volume (mm^3^) | | | | | | | |
| --- | --- | --- | --- | --- | --- | --- | --- | --- |
| 7 | 99.3 | 102.9 | 86.2 | 112.1 | 129.3 | 84.9 | 90.7 | 100.1 |
| 9 | 94.0 | 102.4 | 154.1 | 112.8 | 131.0 | 131.8 | 105.3 | 143.3 |
| 11 | 104.1 | 135.7 | 265.9 | 134.0 | 288.3 | 253.7 | 150.5 | 155.9 |
| 14 | 177.9 | 290.6 | 411.1 | 207.9 | 677.4 | 352.8 | 317.6 | 395.9 |
| 16 | 262.6 | 637.9 | 859.7 | 278.0 | 1389.5 | 440.9 | 499.9 | 500.1 |
| 18 | 441.6 | 1089.8 | 1521.1 | 492.4 | 2269.4 | 471.4 | 810.9 | 786.2 |
| 20 | 898.0 | 1417.3 | 1288.9 | 1376.0 | 1578.0 | 1342.0 | 1122.6 | 879.0 |

| Days | PCC0208025 60 mg/kg, Tumor volume (mm^3^) | | | | | | | |
| --- | --- | --- | --- | --- | --- | --- | --- | --- |
| 7 | 88.0 | 80.2 | 120.2 | 108.0 | 102.8 | 96.6 | 115.1 | 94.6 |
| 9 | 95.3 | 125.7 | 211.1 | 171.9 | 118.3 | 194.9 | 123.1 | 102.4 |
| 11 | 229.3 | 292.5 | 364.6 | 336.0 | 181.3 | 314.7 | 170.3 | 156.3 |
| 14 | 424.1 | 471.2 | 721.6 | 762.9 | 310.1 | 446.5 | 215.6 | 248.3 |
| 16 | 526.4 | 522.4 | 867.5 | 831.7 | 448.6 | 575.4 | 300.5 | 301.9 |
| 18 | 779.5 | 838.2 | 1307.9 | 1195.7 | 630.2 | 795.1 | 427.2 | 419.6 |
| 20 | 843.3 | 1382.8 | 1322.0 | 1565.0 | 743.5 | 1014.6 | 613.5 | 621.2 |
