# Supplementary material for: PCC0208025 (BMS202), a small molecule inhibitor of PD-L1, produces an antitumor effect in B16-F10 melanoma-bearing mice
Source: PLoS One. 2020 Mar 26;15(3):e0228339. doi: 10.1371/journal.pone.0228339 (PMC7098565; doi:10.1371/journal.pone.0228339)
Supplement: S5 Table — B16-F10 tumors mice were administrated by oral gavage with PCC0208025 at 30 mg/kg or 60 mg/kg, twice daily. On day 20, the tumors were removed and weighed. (DOCX) [file pone.0228339.s008.docx]

| Tumor weight (mg) | | |
| --- | --- | --- |
| Control | PCC0208025 30 mg/kg | PCC0208025 60 mg/kg |
| 2369.0 | 885.2 | 1058.9 |
| 2829.0 | 1067.4 | 1055.7 |
| 2993.6 | 1578.0 | 1362.0 |
| 1528.0 | 675.2 | 1678.4 |
| 1249.0 | 1897.0 | 788.5 |
| 2269.1 | 910.0 | 1049.4 |
| 2058.9 | 1479.0 | 652.6 |
| 724.6 | 1094.1 | 524.1 |
