# Supplementary material for: PCC0208025 (BMS202), a small molecule inhibitor of PD-L1, produces an antitumor effect in B16-F10 melanoma-bearing mice
Source: PLoS One. 2020 Mar 26;15(3):e0228339. doi: 10.1371/journal.pone.0228339 (PMC7098565; doi:10.1371/journal.pone.0228339)
Supplement: S6 Table — Before all the mice with tumors were decapitated, the blood samples from orbital venous sinus were collected into tubes with heparin for plasma preparation. Plasma IFN-γ level was determined by using mice ELISA kit. (DOCX) [file pone.0228339.s009.docx]

| IFN-γlevel in plasma (pg/ml) | | |
| --- | --- | --- |
| Control | PCC0208025 30 mg/kg | PCC0208025 60 mg/kg |
| 524.1 | 1257.4 | 1678.5 |
| 428.3 | 869.8 | 1589.6 |
| 479.5 | 713.3 | 1467.7 |
| 683.3 | 967.6 | 1256.8 |
| 354.9 | 1189.8 | 1756.2 |
| 689.8 | 1546.3 | 1476.1 |
