# Supplementary material for: PCC0208025 (BMS202), a small molecule inhibitor of PD-L1, produces an antitumor effect in B16-F10 melanoma-bearing mice
Source: PLoS One. 2020 Mar 26;15(3):e0228339. doi: 10.1371/journal.pone.0228339 (PMC7098565; doi:10.1371/journal.pone.0228339)
Supplement: S7 Table — T cell subsets were counted by flow cytometry from tumors in B16-F10-bearing mice. The percentage of CD3+, CD3+CD8+, CD8+IFN-γ+, CD3+CD4+, and CD4+CD25+CD127low/- T cells were presented. (DOCX) [file pone.0228339.s010.docx]

|  | Control | | | | | |
| --- | --- | --- | --- | --- | --- | --- |
| % CD3^+^ of lymphocyte | 52.45 | 42.85 | 40.80 | 55.16 | 50.54 | 47.77 |
| % CD4^+^ of CD3^+^ | 35.99 | 44.12 | 47.22 | 40.10 | 42.55 | 49.16 |
| % CD8^+^ of CD3^+^ | 39.12 | 28.54 | 29.97 | 36.71 | 27.99 | 31.98 |
| % Treg of CD4^+^ | 40.94 | 38.65 | 39.51 | 38.76 | 40.94 | 35.75 |
| % CD8^+^IFN-γ of CD3D^+^CD8^+^ | 18.13 | 15.82 | 15.60 | 15.91 | 15.63 | 23.51 |

|  | PCC0208025 30 mg/kg | | | | | |
| --- | --- | --- | --- | --- | --- | --- |
| % CD3^+^ of lymphocyte | 58.95 | 61.13 | 52.42 | 62.48 | 58.92 | 54.72 |
| % CD4^+^ of CD3^+^ | 38.97 | 39.86 | 36.50 | 29.65 | 37.02 | 40.10 |
| % CD8^+^ of CD3^+^ | 38.80 | 39.05 | 45.77 | 42.49 | 39.23 | 36.71 |
| % Treg of CD4^+^ | 26.25 | 33.66 | 35.98 | 35.44 | 36.86 | 31.41 |
| % CD8^+^IFN-γ of CD3D^+^CD8^+^ | 27.98 | 25.40 | 24.84 | 24.39 | 22.29 | 22.23 |

|  | PCC0208025 60 mg/kg | | | | | |
| --- | --- | --- | --- | --- | --- | --- |
| % CD3^+^ of lymphocyte | 72.64 | 70.58 | 61.04 | 62.21 | 61.98 | 64.77 |
| % CD4^+^ of CD3^+^ | 36.42 | 31.49 | 39.77 | 27.21 | 38.87 | 37.34 |
| % CD8^+^ of CD3^+^ | 40.72 | 50.51 | 43.82 | 50.59 | 42.04 | 42.77 |
| % Treg of CD4^+^ | 32.63 | 30.22 | 32.76 | 31.72 | 28.26 | 23.46 |
| % CD8^+^IFN-γ of CD3D^+^CD8^+^ | 30.80 | 35.94 | 30.46 | 29.24 | 28.35 | 27.49 |
