# Supplementary material for: PCC0208025 (BMS202), a small molecule inhibitor of PD-L1, produces an antitumor effect in B16-F10 melanoma-bearing mice
Source: PLoS One. 2020 Mar 26;15(3):e0228339. doi: 10.1371/journal.pone.0228339 (PMC7098565; doi:10.1371/journal.pone.0228339)
Supplement: S8 Table — After a single dose of 60 mg/kg PCC0208025, PCC0208025 concentrations were detected in plasma and tumors at 1h, 3h and 8h, respectively. (DOCX) [file pone.0228339.s011.docx]

| Time | PCC0208025 Concentration in Plasma (nM) | | | | | PCC0208025 Concentration  in Tumor (nmol/kg) | | | | |
| --- | --- | --- | --- | --- | --- | --- | --- | --- | --- | --- |
| 1h | 3.80 | 4.30 | 4.97 | 4.53 | 4.13 | 207.3 | 154.8 | 139.1 | 136.3 | 167.3 |
| 3h | 4.16 | 4.09 | 3.56 | 3.78 | 3.59 | 206.5 | 218.5 | 178.7 | 206.5 | 185.3 |
| 8h | 3.02 | 2.96 | 3.50 | 3.14 | 3.26 | 146.7 | 123.3 | 103.8 | 133.2 | 132.4 |
