# Supplementary material for: Correction: PCC0208025 (BMS202), a small molecule inhibitor of PD-L1, produces an antitumor effect in B16-F10 melanoma-bearing mice
Source: PLoS One. 2021 Apr 28;16(4):e0251020. doi: 10.1371/journal.pone.0251020 (PMC8081209; doi:10.1371/journal.pone.0251020)
Supplement: S1 File — (PDF) [file pone.0251020.s001.pdf]

PLOS ONE

RESEARCH ARTICLE

PCC0208025 (BMS202), a small molecule inhibitor of PD-L1, produces an antitumor effect in B16-F10 melanoma-bearing mice

Zhengping Hu[1], Pengfei Yu[2], Guangying Du[3], Wenyan Wang[3], Haibo Zhu[2], Ning Li[2], Huijuan Zhao[2], Zhaoju Dong[2], Liang Ye[2]*, Jingwei Tian[3]*

1 Medicine & Pharmacy Research Center, Binzhou Medical University, Yantai, Shandong, China, **2** School of Public Health and Management & Institute of Toxicology, Binzhou Medical University, Yantai, Shandong, China, **3** School of Pharmacy, Key Laboratory of Molecular Pharmacology and Drug Evaluation (Yantai University), Ministry of Education, Collaborative Innovation Center of Advanced Drug Delivery System and Biotech Drugs in Universities of Shandong, Yantai University, Yantai, China

* project0088@hotmail.com (LY); tianjeanswest@gmail.com (JT)

Check for updates

OPEN ACCESS

Citation: Hu Z, Yu P, Du G, Wang W, Zhu H, Li N, et al. (2020) PCC0208025 (BMS202), a small molecule inhibitor of PD-L1, produces an antitumor effect in B16-F10 melanoma-bearing mice. PLoS ONE 15(3): e0228339. https://doi.org/10.1371/journal.pone.0228339

Editor: Fabrizio Mattei, Istituto Superiore di Sanità, ITALY

Received: July 14, 2019

Accepted: January 13, 2020

Published: March 26, 2020

Copyright: © 2020 Hu et al. This is an open access article distributed under the terms of the Creative Commons Attribution License, which permits unrestricted use, distribution, and reproduction in any medium, provided the original author and source are credited.

Data Availability Statement: ***NOTE TO PA AT ACCEPT: Ask authors to make the data publicly available**** Data will be available after the acceptance.

Funding: This work was supported by the National Natural Science Foundation of China (grant no. 81473188), the Natural Science Foundation of Shandong Province, China (No. ZR2014HM091), Natural Science Foundation of Shandong Province (No. ZR2016HP15), and the Scientific Research Foundation of Binzhou Medical College, Shandong

Abstract

The increased PD-L1 expression induces poorer prognosis in melanoma. The small molecule inhibitors of PD-1/PD-L1 pathways have been an encouraging drug development strategy because of good affinity and oral bioavailability without immunogenicity and immunotoxicities of PD-1/PD-L1 antibodies. In this study, we studied the effects of PCC0208025 (BMS202), a small molecule inhibitor of PD-L1, on PD-1/PD-L1 binding and the cytokines secretion in human $CD3^+$ cells *in vitro*. We also investigated the antitumor and immunomodulatory activity of PCC0208025 and the pharmacokinetics properties in B16-F10 melanoma-bearing mice. The results showed that PCC0208025 inhibited the PD-1/PD-L1 proteins binding, and rescued PD-L1-mediated inhibition of IFN-γ production in human $CD3^+$ T cells *in vitro*. Furthermore, in B16-F10 melanoma-bearing mice, PCC0208025 presented the antitumor effects, enhanced IFN-γ levels in plasma, increased the frequency of $CD3^+CD8^+$ T and $CD8^+$IFN-γ^+ T and the ratios of $CD8^+$/Treg, and deceased the $CD4^+CD25^+CD127^{low/-}$ (Treg) number in tumor. Pharmacokinetics study found that PCC0208025 was absorbed and distributed into the tumors with much higher concentrations than those of the blockade against PD-1/PD-L1 binding. Our work suggests that PCC0208025 exhibited anti-tumor effects through inhibiting Treg expansion and increasing cytotoxic activity of tumor-infiltrating $CD8^+$ T cells by the blockade of PD-1/PD-L1 binding, which may provide the pharmacological basis to develop small molecule inhibitors of PD-1/PD-L1 binding for PCC0208025 as a lead compound.

Introduction

Melanoma is the main cancer which caused skin cancer-related deaths [1,2]. A 5-year survival rate is below 10% with traditional therapies [1–3]. Recently, immunotherapies have become the standard treatment regimens for the melanoma patients [2–4].

In the tumor microenvironment, the activated PD-1/B7-H1 (programmed cell death 1/ PD-L1) signaling pathway makes T lymphocytes functionally inactivated [2,5], which decreases the anti-tumor activity of T cells. So far, the PD-1 antibodies (pembrolizumab and

Province, China (No. BY2013KYQD21). The funders had no role in study design, data collection and analysis, decision to publish, or preparation of the manuscript.

Competing interests: Luye Pharma provided the cell culture room for us to cultrue B16-F10 and CT26.WT tumor cells, and human CD3+ cells. This does not alter our adherence to PLOS ONE policies on sharing data and materials.

nivolumab), have been approved for the treatment of the advanced melanoma [2,6,7]. In addition, the PD-1 receptor ligand (PD-L1) antibodies (BMS-936559 and atezolizumab) have been studied for treating melanoma in preclinical mouse models and clinical trials [2,8].

However, the monoclonal antibodies has some disadvantages such as the immunogenicity, lower bioavailability, poor solid tumor tissue distribution and difficult controlled pharmacokinetics, and thus antibody related toxicities [9–13]. In contrast, the small molecules posses good affinity, specificity and oral bioavailability without the immunogenicity. The chemical inhibitors for targeting the PD-1/PD-L1 pathway or PD-1/PD-L1 interaction, such as small molecules, macrocyclic peptides, peptides and peptidomimetics, have been reported [9,14]. Several small molecule inhibitors of PD-L1 from Bristol-Myers Squibb (BMS) have been studied with the good blockade activity of PD-1/PD-L1 binding [10,13,15]. One of these agents, BMS-202 (N-(2-{[2-Methoxy-6-(2-methyl-biphenyl-3-ylmethoxy)-pyridin-3-ylmethyl]-amino}-ethyl)-acetamide), was resynthesized and renamed as PCC0208025 in our lab. BMS202 inhibits PD-1 and PD-L1 binding with an IC_{50} of 0.018 μM in an HTRF binding assay [13,16], which was superior to other BMS compounds according to the BMS patent (page 172) [16]. However, the patent did not include any additional biological data, and a thorough review of the literature revealed that no other in vitro and in vivo data has been reported for PCC0208025 (BMS202), indicating further activity validation in vivo and in vitro for PCC0208025 (BMS202) is warranted [9,13].

PCC0208025 is a PD-L1 inhibitor with a chemical structure as shown in Fig 1. In the present study, we investigated the potential effects of PCC0208025 on the PD-1/PD-L1 complex formation and the cytokines secretion in human $CD3^+$ T cells *in vitro*. We also studied the antitumor and the immunomodulatory activity of PCC0208025, and pharmacokinetics properties in B16-F10 melanoma-bearing mice.

Materials and methods

Drug and reagents

PCC0208025 was synthetized as a white solid by biochempartner company (Shanghai, China) with the molecular formulae of $C_{25}H_{29}N_3O_3$. ^{1}H NMR (300 MHz, DMSO-d_6) δ 7.84 (s, 1H), 7.63 (d, J = 5.7 Hz, 1H), 7.47–7.36 (m, 4H), 7.31–7.17 (m, 4H), 6.43 (d, J = 6 Hz, 1H), 5.41 (s, 2H), 3.90 (s, 3H), 3.59 (s, 2H), 3.15–3.11 (m, 2H), 2.55 (t, 2H), 2.21 (s, 3H), 1.79 (s, 3H). MS (m/z) 420.3 $[M+H]^+$. HPLC purity 98.62%.

The recombinant human PD-L1 protein (ab167713) was bought from Abcom company (USA) for studying the effects of PCC0208025 on IFN-γ secretion in human $CD3^+$ cells *in vitro*. The human anti-PD-L1 antibody (BMS-936559) was expressed by Crown BioScience company (Suzhou, China). The anti-$CD3^+$ antibody (MAB100, Clone # UCHT1) and anti-

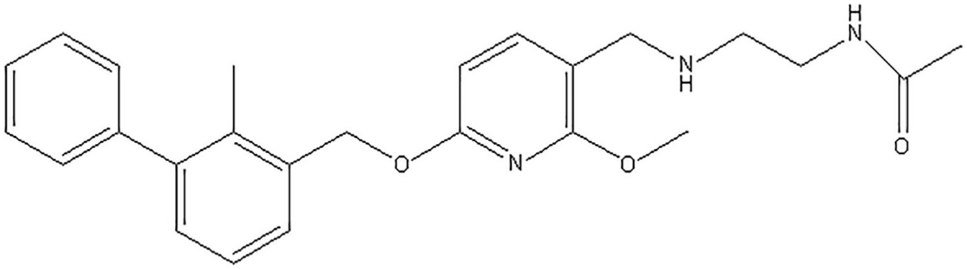

Fig 1. The structure of PCC0208025.

https://doi.org/10.1371/journal.pone.0228339.g001

$CD8^+$ antibody (MAB342-100, Clone # 37407) were provided by R&D Systems (USA). Human interferon gamma (IFN-γ) ELISA Kit (DIF50) and mice IFN-γ ELISA kit (MIF00) were purchased from R&D Systems (USA). Human PD-1/PD-L1 binding assay kit (Part # 64ICP01PEG) was obtained from Cisbio company (Shanghai, China), which mainly contains some reagents such as Tag2-PD-1, Tag1-PD-L1, anti-Tag1-EuK and anti-Tag2-XL665. BV421 anti-mouse CD3 (100228), BV510 anti-mouse CD4 (100449), FITC anti-mouse CD8 (100706), BV605 anti-mouse CD25 (102036) and APC anti-mouse CD127 (135012) were provided by BioLegend company (Canada). PE anti-mouse IFN-γ (12-7311-82) and the Intracellular Fixation & Permeabilization Buffer Set (88–8824) were obtained from Ebioscience company (USA). Leukocyte Activation Cocktail with GolgiPlug (550583) and FACS Staining Buffer (554656) was provided by BD Pharmingen Inc. (USA).

Cytotoxicity of PCC0208025 to tumor cells and human $CD3^+$ cells in vitro

Mouse melanoma cell line B16-F10 (ATCC® Number: CRL-6475™) and mouse cell line CT26. WT (TCM37) were obtained from Cell Culture Center of the Institute of Basic Medical Sciences, Chinese Academy of Medical Sciences. The cells were maintained in DMEM supplemented with 10% (v/v) heat-inactivated fetal bovine serum (FBS) in a humidified 5% CO_2 atmosphere at 37 ˚C. Tumor cells were plated in 96-well plate with a density of 5×10^3 cells/well in 100 μL. After 24 h incubation, the cells were exposed to PCC0208025 with the final concentration of 2.5, 5, 10, 20, 40 an 80 μM in the final volume of 200 μL medium and cultured at 37 ˚C in a humidified atmosphere for 24 h and 48 h. 20 μL of 3-(4,5-dimethylthiazol-2-yl)-2,5-diphenyltetrazolium bromide (MTT, 5 mg/ml) was added to each well and incubated for additional 4 h at 37 ˚C. The medium was subsequently discarded, and 150 μL of DMSO was added to dissolve the formazan crystals [17]. The absorbance was measured at 570 nm using a Molecular Devices Spectra Max M5 (Molecular Devices, USA).

Human $CD3^+$ cells with the purity above 95% were provided by LDEBIO company (Guangzhou, China). $CD3^+$ cells were plated in the 96-well plate with a density of 1×10^5 cells/well in 100 μL of DMEM medium containing 10% FBS. The 50 μL of anti-CD3 antibody and anti-CD28 antibody (aCD3 and aCD28, final concentration of 1 μg/mL for each) were added into 96-well plate. Then the $CD3^+$ cells were exposed to PCC0208025 with the final concentration of 2.5, 5, 10, 20, 40 an 80 μM in the final volume of 200 μL medium, and cultured at 37 ˚C in a humidified atmosphere for 72 h. CellTiter-Glo (CTG) reagent was added into the wells to measure $CD3^+$ cell viability.

Effects of PCC0208025 on IFN-γ expression in human $CD3^+$ cells in vitro

Human $CD3^+$ cells were plated in the 96-well plate with a density of 1×10^5 cells/well in 100 μL of DMEM containing 10% FBS. The 50 μL of aCD3 and aCD28 (final concentration of 1 μg/mL for each) were added into 96-well plate without or with 50 μL of human PD-L1 protein (final concentation of 10 nM). Then 50 μL of BMS-936559 or PCC0208025 solution with the final concentrations of 0.01, 0.1, 1 and 10 μM were added to the wells and cultured at 37 ˚C in a humidified atmosphere for 72 h. The supernatants were collected for detection of IFN-γ by using human IFN-γ ELISA Kit.

Homogeneous Time-Resolved Fluorescence (HTRF) binding assay

In HTRF assay, the binding of Tag2-PD-1 and Tag1-PD-L1 is detected by anti-Tag1-EuK (HTRF donor) and anti-Tag2-XL665 (HTRF acceptor). Compounds or antibodies blocking PD-1/PD-L1 complex formation reduce the HTRF signal. Briefly, Tag2-PD-1 (20 nM final), PCC0208025 (0.15, 0.46, 1.37, 4.12, 12.35, 37.04, 111.11, 333.33 and 1000 nM, final) or BMS-

936559 (0.002, 0.006, 0.024, 0.10, 0.39, 1.56, 6.25, 25 and 100 nM final), and Tag1-PD-L1 (2 nM final) were diluted in Binding Domain diluent buffer, and added one after the other to a 384-well plate for a total volume of 10 μl. After 15 min pre-incbation, an addition of 10 μl of detection reagent containing 1.83 nM anti-Tag1-EuK and 66.7 nM anti-Tag2-XL665 prepared in Binding Domain Detection buffer #1 was added into the assay well. The signals (665 nm/620 nm ratio) were obtained on Tecan M200 PRO. HTRF ratio = $(OD_{665\ nm}/OD_{620\ nm}) \times 10^4$.

Animals

Male C57BL/6NCrl mice (4–5 weeks old; purchased from Vital River Laboratory Animal Technology Co., Ltd) were used for *in vivo* experiments. Animals were maintained under controlled environment at 25 ˚C on a 12-h light/dark cycle, which was free access to food and water. This experiment was approved by the Ethics Committee of Binzhou Medical University (No. 013 in 2014 for Animal Ethics Approval). The local legislation regarding the ethics of animal experimentation and the guidelines for the care and use of laboratory animals were followed in all animal procedures. All mice were intraperitoneally injected with 10 mg/kg of pentobarbital sodium to induce anesthesia before the surgery.

In vivo tumor isograft model and dosing regimen

B16-F10 tumors were established by injecting 1×10^5 cells mixed with matrigel into the dorsal area of male mice [18–20]. On 2rd day, the mice bearing tumors were randomly divided into three groups (12/each group). Mice were administrated by oral gavage with PCC0208025 at 30 mg/kg or 60 mg/kg with a volume of 0.1 ml/10 g, twice daily. Control mice were given the same volume of saline. On days 7, 9, 11, 14, 16, 18 and 20, tumor dimensions were measured. Tumor volumes were calculated according to the following formula: volume (mm^3) = $0.5 \times$ length (mm) $\times$ width (mm) $\times$ width (mm). On day 20, all the mice were decapitated between 9:00 a.m. and 11:00 a.m.. The tumors were obtained. And the inhibition rate (IR) of tumor growth was calculated by the following formula: IR (%) = $[(A - B)/A] \times 100$, where A and B were the mean tumor weight in the control and treatment groups, respectively.

Measurements for plasma IFN-γ level in melanoma-bearing mice

Before all the mice were decapitated, the blood samples from orbital venous sinus were collected into tubes with heparin for plasma preparation. These samples were stored at -80˚C for tests. Plasma IFN-γ level was determined by using mice ELISA kit according to the manufacturer's instructions [21].

Flow cytometry analyses for T lymphocytes in tumors from melanoma-bearing mice

At the end of the experiment (day 20), tumor tissues were harvested and 6 out of 12 were randomly selected according to the tumor weight in each group for flow cytometric analysis. Single cell suspensions were prepared and a Ficoll-Hypaque purification step was carried out for the tumor-derived cell suspension [22]. After the cells were washed twice with PBS and resuspended in DMEM supplemented with 1% FBS. 100 μL of cell suspension per tube, containing 2×10^5 cells, was stimulated with 200 μL of Leukocyte Activation Cocktail with GolgiPlug in a 37˚C humidified CO_2 incubator for 6 h. Following activation, the cells were harvested and washed with FACS Staining Buffer, and used for antibody staining for 30 min at 4˚C by using BV421 anti-mouse CD3, BV510 anti-mouse CD4, FITC anti-mouse CD8, BV605 anti-mouse CD25 and APC anti-mouse CD127. These tubes were centrifuged at 1200 rpm for 5 min and

the supernatant were discarded, followed by an addition of 200 μl of the intracellular fixation buffer to each tube and incubating for 30 min at room temperature. The cells were washed twice with the permeabilization buffer and resuspended in the permeabilization buffer. PE anti-mouse IFN-γ antibody was added and incubated for 30 min in the dark at 4˚C. The cell counts for the $CD3^+$, $CD3^+CD4^+$, $CD3^+CD8^+$, $CD4^+CD25^+CD127^{low/-}$ (Treg) and $CD8^+$IFN-γ^+ T lymphocytes were assessed via flow cytometry (BD FACSCanto II, California, USA). Finally, the ratios of $CD8^+$/Treg were calculated.

Pharmacokinetics of PCC0208025 in plasma and tumor from melanoma-bearing mice

In order to know the plasma and tumor concentrations of PCC0208025, 15 male C57BL/6NCrl mice were used to establish B16-F10 melanoma-bearing model according to the above method. When the tumors grew with the volume of about 1000 mm^3, these mice were administrated by oral gavage with single dose of PCC0208025 at 60 mg/kg. At 1h, 3h and 8h after the dosing, 5 mice were decapitated, respectively, for collecting plasma and tumor tissues. All plasma samples were centrifuged for 10 min at 3000 g, separated and stored at -20 ˚C for followed analysis. The tumor tissues were homogenated in water (w/v = 1:4). The LC-MS/MS system consisted of an Agilent 1100 series HPLC system (Agilent Technologies, Waldbronn, USA) and a TSQ Quantum Assess tandem mass spectrometer (Thermo Electron Corporation, San Jose, CA, USA) equipped with ESI ion source operation in the positive mode. Data acquisition and processing were accomplished using the Xcaliber workstation. The chromatography separation was performed on a Waters symmetry CLU columns (150 × 2.1 mm i.e., 3.5 am, Warders, USA) with the flow rate of 0.2 ml/min in isocratic elution. The mobile phase consisted of acetonitrile-methanol-water (50:30:20, v/v/v) containing 0.1 mm ammonium acetate and 0.01% glacial acetic acid. The selective reactions monitoring mode was used to detect the analyses. The precursor/product transitions were at m/z 420.2–317.9 for PCC0208025, and m/z 427.1–207.0 for paliparidone (IS). The spray voltage was 4 kv. Sheath gas and auxilinry gas were 30 and 5 psi, respectively. The capillary temperature was 350 ˚C and argon gas pressure was 1.5 milli-Torr. The collision induced dissociation voltage was 15 V for PCC0208025 and 28 V for IS. The plasma and tumor drug concentration-time curves were displayed as PCC0208025 concentration in μM and μmol/kg, respectively, at various time points.

Statistical analyses

The statistical analyses were performed using one-way ANOVA, followed by least significant difference (LSD) post hoc test in SPSS software (V 16.0). $P < 0.05$ was considered statistically significant.

Results

Cytotoxicity of PCC0208025 to tumor cells and human $CD3^+$ cells in vitro

In order to investigate cytotoxicity of PCC0208025, both tumor cells and $CD3^+$ cells were exposed to different concentrations of PCC0208025. The results in Table 1 showed that IC_{50} were above 10.0 μM to mice tumor cells and human $CD3^+$ cells, which indicates PCC0208025 possesses low cytotoxicity *in vitro*.

Table 1. Effects of PCC0208025 on several cell lines viability were determined by MTT assay or CTG assay.

Time	IC_{50}(μM)		
	B16F10	CT26	Human $CD3^+$ cells
24 h	32.1 ± 2.3	21.9 ± 2.9	N/A
48 h	23.5 ± 3.2	15.3 ± 3.4	N/A
72 h	N/A	N/A	10.3 ± 1.3

N/A, not assay. IC_{50} values were calculated using Graph Pad Prism v 5.0. The results were presented as mean ± S.D (n = 3).

https://doi.org/10.1371/journal.pone.0228339.t001

HTRF binding assay

In order to detect the inhibitory effects of PCC0208025 against PD-1 and PD-L1 binding, HTRF binding assay was conducted. Anti-PD-L1 antibody BMS-936559 and the compound PCC0208025 showed high activity with IC_{50} of 0.54 nM and 235 nM, respectively (Fig 2).

Effects of PCC0208025 on IFN-γ level in human $CD3^+$ cells in vitro

Cytokines play an important role in the immune response, and we investigated the effects of PCC0208025 on the production of cytokine IFN-γ in human $CD3^+$ cells *in vitro*. As shown in Fig 3, combined aCD3 and aCD28 significantly increased the IFN-γ expression compared with

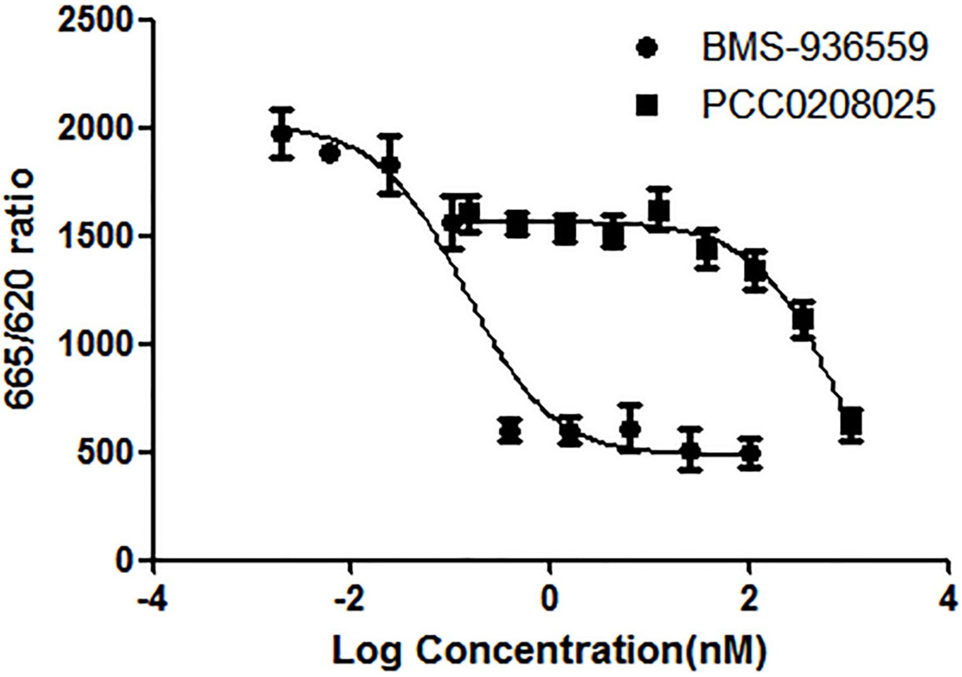

Fig 2. The inhibitory effects of PCC0208025 against PD-1 and PD-L1 binding. In HTRF assay, Tag2-PD-1 (20 nM final), PCC0208025 (0.15, 0.46, 1.37, 4.12, 12.35, 37.04, 111.11, 333.33 and 1000 nM, final) or BMS-936559 (0.002, 0.006, 0.024, 0.10, 0.39, 1.56, 6.25, 25 and 100 nM final), and Tag1-PD-L1 (2 nM final) were diluted in Binding Domain diluent buffer, and added one after the other to a 384-well plate for a total volume of 10 μl. After 15 min pre-incubation, an addition of 10 μl of detection reagent containing 1.83 nM anti-Tag1-EuK and 66.7 nM anti-Tag2-XL665 was added into the assay well. The signals (665 nm/620 nm ratio) were obtained on Tecan M200 PRO. HTRF ratio = $(OD_{665\ nm}/OD_{620\ nm}) \times 10^4$. The results showed that BMS-936559 and PCC0208025 presented high activity with IC_{50} of 0.54 nM and 235 nM.

https://doi.org/10.1371/journal.pone.0228339.g002

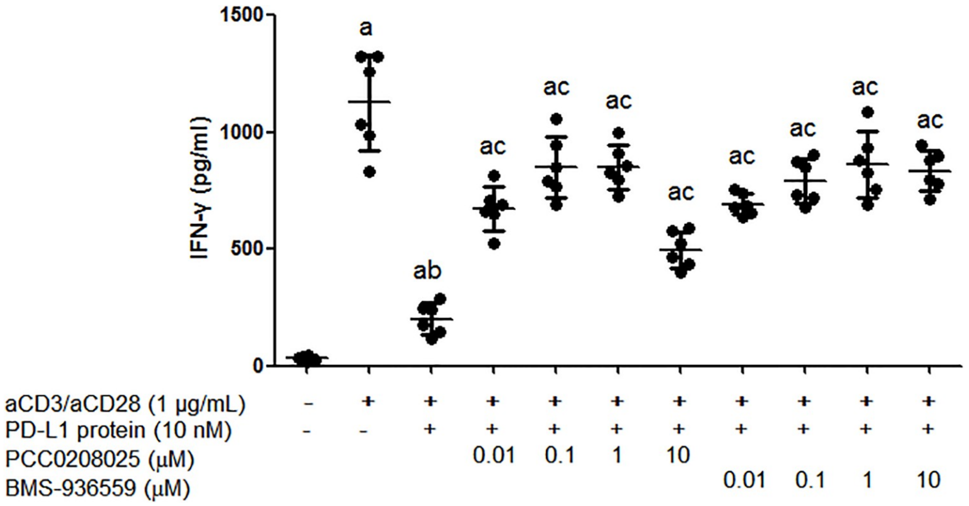

Fig 3. Effects of PCC0208025 on IFN-γ secreted by $CD3^+$ cells *in vitro*. Human $CD3^+$ cells were plated in the 96-well plate with a density of 1×10^5 cells/well in 100 μL of DMEM containing 10% FBS. The 50 μL of aCD3 and aCD28 (final concentration of 1 μg/mL for each) were added into 96-well plate without or with 50 μL of human PD-L1 protein (final concentation of 10 nM). Then 50 μL of BMS-936559 or PCC0208025 solution with the final concentrations of 0.01, 0.1, 1 and 10 μM were added to the wells and cultured for 72 h. The supernatants were collected for detection of IFN-γ by using human IFN-γ ELISA Kit. The results were presented as mean ± SD (n = 6). $^aP < 0.05$ compared with medium control group. $^bP < 0.05$ compared with aCD3/aCD28 control group. $^cP < 0.05$ compared with aCD3/aCD28 and PD-L1 group.

https://doi.org/10.1371/journal.pone.0228339.g003

medium control (each treatment $P < 0.05$, n = 6), which was significantly decreased by human PD-L1 protein (each treatment $P < 0.05$, n = 6). However, anti-PD-L1 antibody BMS-936559 and the compound PCC0208025 from 0.01 to 1 μM markedly rescued PD-L1-mediated inhibition of IFN-γ production (each treatment $P < 0.05$, respectively, n = 6).

Effects of PCC0208025 on the tumor growth in B16-F10-bearing mice

We investigated the anti-cancer activities of PCC0208025 in B16-F10-bearing mice. We found that treatment with PCC0208025 at 30 mg/kg and 60 mg/kg significantly decreased tumor weight ($P < 0.05$, n = 8) and tumor volumes (day 20, $P < 0.05$, n = 8) compared with the control group (Fig 4A and 4B). According to tumor weight, 30 mg/kg and 60 mg/kg of PCC0208025 presented the IR of 30.3% and 50.1%, respectively.

Effects of PCC0208025 on plasma IFN-γ level in melanoma-bearing mice

To investigate the effects of PCC0208025 on the immune function in B16-F10-bearing mice, we detected the plasma IFN-γ level using mice ELISA kit. As shown in Fig 5, PCC0208025 of 30 and 60 mg/kg markedly elevated plasma IFN-γ levels compared with the control group (each treatment $P < 0.05$, n = 6).

Flow cytometry analyses for T lymphocytes in tumors

To investigate the effects of PCC0208025 on the cellular immunity, the cell counts for $CD3^+$, $CD3^+CD4^+$, $CD3^+CD8^+$, $CD4^+CD25^+CD127^{low/-}$ and $CD8^+$IFN-γ^+ T lymphocytes in tumor were determined by flow cytometry. As shown in Figs 6 and 7, the percentage of $CD3^+$, $CD3^+CD8^+$ or $CD8^+$IFN-γ^+ T cells was markedly increased in PCC0208025 treatment groups compared with the control group (each treatment $P < 0.05$ for each T cell, respectively, n = 6).

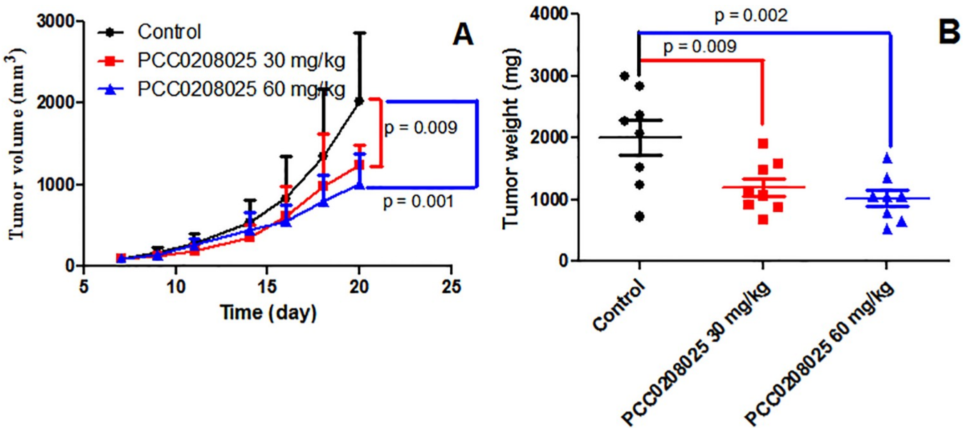

Fig 4. Effects of PCC0208025 on tumor growth in B16-F10 tumor isograft model. B16-F10 tumors were established by injecting 1×10^5 cells mixed with matrigel into the dorsal area of male mice. On 2rd day, the mice bearing tumors were randomly divided into three groups. Mice were administrated by oral gavage with PCC0208025 at 30 mg/kg or 60 mg/kg with a volume of 0.1 ml/10 g, twice daily. Control mice were given the same volume of saline. On days 7, 9, 11, 14, 16, 18 and 20, tumor dimensions were measured. On day 20, all the mice were decapitated between 9:00 a.m. and 11:00 a.m.. The tumors were obtained. And the inhibition rate (IR) of tumor growth was calculated. (A) PCC0208025 had tumor inhibition effects in tumor volume compared with control group ($P < 0.05$, n = 8) on day 20. (B) PCC0208025 had significant tumor inhibition effects in tumor weight compared with control group ($P < 0.05$, n = 8). The results were presented as mean ± SD. $^*P < 0.05$ compared with control group.

https://doi.org/10.1371/journal.pone.0228339.g004

And, significant decreases in the percentage of $CD3^+CD4^+$ T cells were observed in PCC0208025 60 mg/kg group compared with the control group ($P < 0.05$, n = 6). However, the percentage of $CD4^+CD25^+CD127^{low/-}$ (Treg) was significantly decreased by 30 mg/kg and 60 mg/kg of PCC0208025 compared with the control group (each treatment $P < 0.05$, respectively, n = 6). Furthermore, in PCC0208025 30 mg/kg and 60 mg/kg groups, the ratios of $CD8^+$/Treg were significantly increased compared with the control group (each treatment $P < 0.05$, respectively, n = 6).

Pharmacokinetics of PCC0208025 in plasma and tumor from melanoma-bearing mice

For determining PCC0208025 pharmacokinetics characteristics in melanoma-bearing mice, the plasma and tumor PCC0208025 concentrations were detected by HPLC-MS. After a single dose of 60 mg/kg, average concentrations were about 4.36, 3.94 and 3.16 nM in plasma at 1h, 3h and 8h, respectively (each time point, n = 5); and about 160.7, 196.7 and 127.3 nmol/kg in tumor at 1h, 3h and 8h, respectively (each time point, n = 5). These data showed PCC0208025 concentrations decreased slowly in plasma and tumor, while tumor tissue obtained higher concentration at 3h (Fig 8).

Discussion

BMS recently disclosed the first non-peptidic small molecule inhibitors against the PD-1/PD-L1 pathway that highlighted the activity in a HTRF binding assay, including PCC0208025 (BMS-202) [16]. Udhwani, T reevaluated 311 PD-L1 ligand inhibitors and the docking results showed that BMS-202 still bind to PD-L1 dimeric structure with the highest affinity [14]. Because of the potential of BMS-202 (PCC0208025) as the lead compound of PD-L1 inhibitor, we further evaluated PCC0208025.

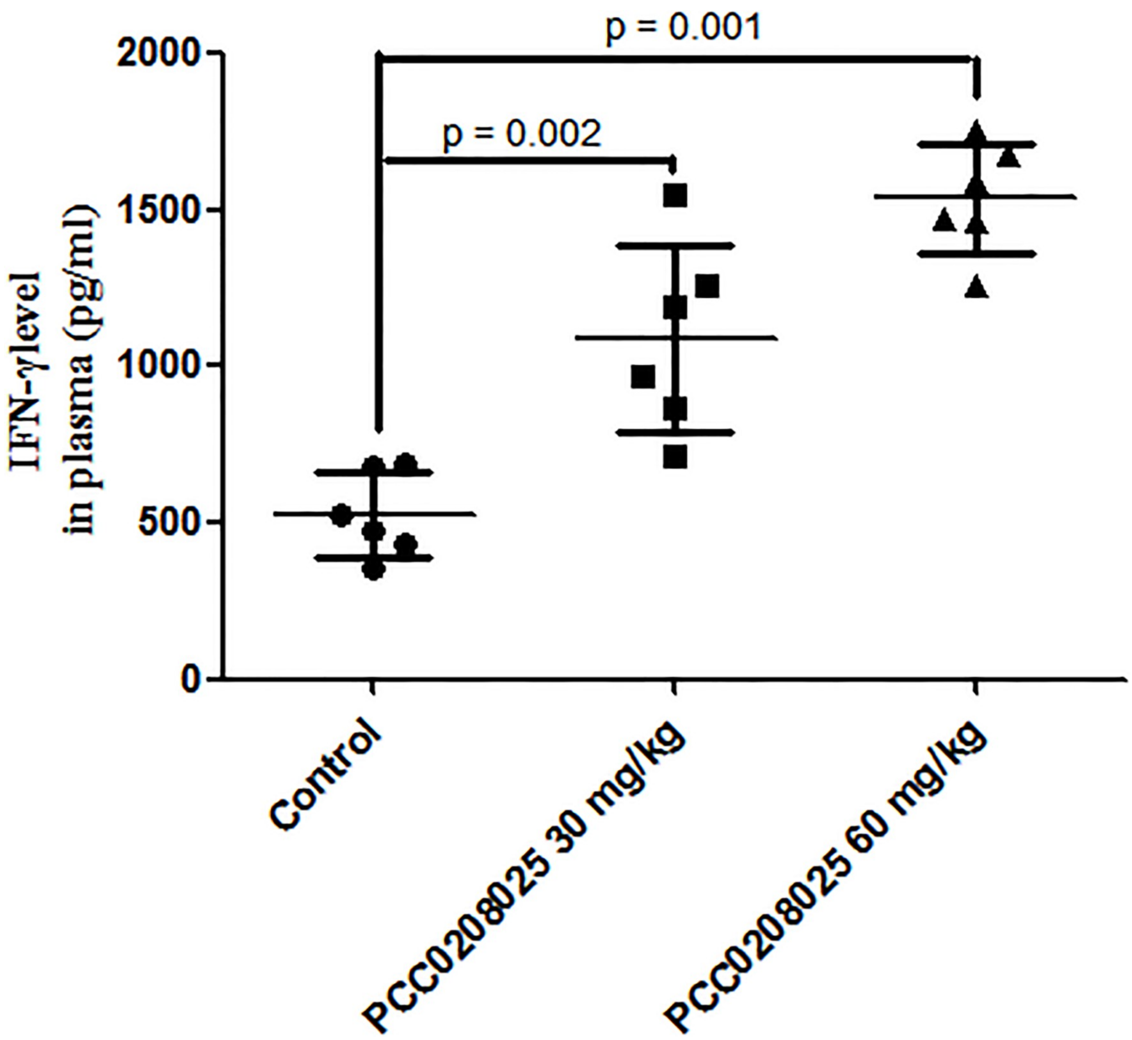

Fig 5. Effects of PCC0208025 on plasma IFN-γ level in B16-F10-bearing mice. Before all the mice with tumors were decapitated, the blood samples from orbital venous sinus were collected into tubes with heparin for plasma preparation. Plasma IFN-γ level was determined by using mice ELISA kit. The increase in plasma IFN-γ levels was observed in 30 mg/kg and 60 mg/kg groups compared with the control group (each treatment $P < 0.05$, respectively, n = 6). The results were presented as mean ± SD. $^{*}P < 0.05$, compared with control group.

https://doi.org/10.1371/journal.pone.0228339.g005

In out study, we found that PCC0208025 neither directly inhibit B16-F10 and CT-26 cells growth *in vitro*, nor killed human $CD3^{+}$ cells at the designed concentration. However, PCC0208025 significantly decreased tumor volumes and tumor weights in B16-F10-bearing mice. These above results demonstrated that PCC0208025 did not directly kill tumor cells, which promoted us to carry out the further experiments to investigate the antitumor mechanisms in melanoma.

The PD-1 and PD-L1 interactions play a critical role in the tumor immune escape by inhibiting effector T cell proliferation, decreasing cytotoxic activity, inducing apoptosis in tumor-infiltrating T cells, and increasing Treg expansion [23]. In our study, HTRF binding assay showed that PCC0208025 presented the inhibition activity against the PD-1 and PD-L1 binding with IC_{50} of 235 nM, which is lower than the results obtained in the literature [13,14]. The different results were due to different HTRF kits. Furthermore, this report proved that PCC0208025 can occlude the PD-1 interaction surface of PD-L1 to form a dimeric protein

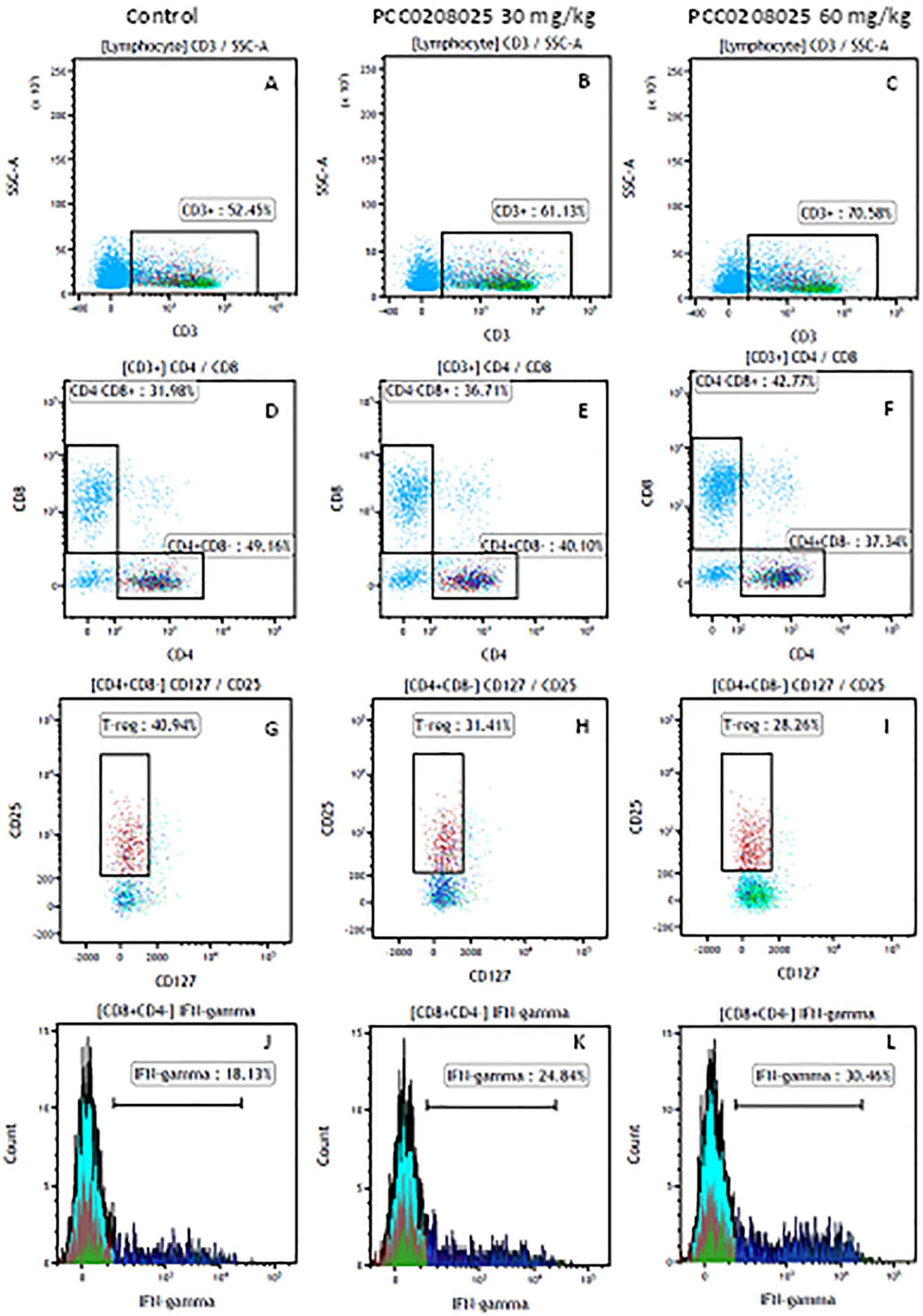

Fig 6. The representative figures for T cell subsets counted by flow cytometry from tumors in B16-F10-bearing mice. The cell counts for $CD3^+$ (A, B and C), $CD3^+CD4^+$ (D, E and F), $CD3^+CD8^+$ (D, E and F), $CD4^+CD25^+CD127^{low/-}$ (G, H and I) and $CD8^+IFN\text{-}\gamma^+$ (J, K and L) T lymphocytes from mouse tumor were determined by flow cytometry.

https://doi.org/10.1371/journal.pone.0228339.g006

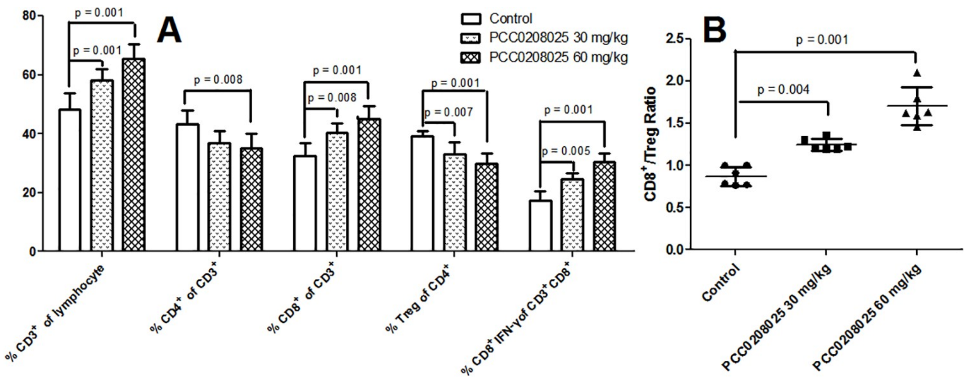

Fig 7. Effects of PCC0208025 on T cell subsets from tumor in B16-F10-bearing mice. The percentage of $CD3^+$, $CD3^+CD8^+$ or $CD8^+IFN\text{-}\gamma^+$ T cells was markedly increased in PCC0208025 groups compared with the control group (each treatment $P < 0.05$ for each T cell, respectively, n = 6) (A). And, The significant decreases in the percentage of $CD3^+CD4^+$ T cells were observed in PCC0208025 60 mg/kg group compared with the control group ($P < 0.05$, n = 6) (A). However, the percentage of $CD4^+CD25^+CD127^{low/-}$(Treg) was significantly decreased by 30 mg/kg and 60 mg/kg of PCC0208025 compared with the control group (each treatment $P < 0.05$, respectively, n = 6) (A). Furthermore, in PCC0208025 30 mg/kg and 60 mg/kg groups, the ratios of $CD8^+$/Treg (B) were significantly increased compared with the control group (each treatment $P < 0.05$, respectively, n = 6). The results were presented as mean ± SD. $^*P < 0.05$ compared with control group.

https://doi.org/10.1371/journal.pone.0228339.g007

complex [13,14], which provides a rationale that PCC0208025 can inhibit the PD-1/PD-L1 signaling pathway in T cells.

In order to evaluate the effects of PCC0208025 on the PD-1/PD-L1 signaling pathway, we investigated the rescue effects of PCC0208025 on the IFN-γ production decrease induced by human PD-L1 protein in human $CD3^+$ T cells *in vitro*. We found that PCC0208025 rescued PD-L1-mediated inhibition of IFN-γ production, which was due to the blockade of PCC0208025 against PD-1/PD-L1 signaling pathway.

The T cells are very important for inducing the immune response to tumor antigens [2]. Our study showed that less $CD3^+CD8^+$ T cells were presented in the untreated B16-F10 tumor, which was closely related with tumor growth. As the result of the PD-1/PD-L1 signaling pathway blockade, PCC0208025 increased the $CD3^+CD8^+$ T cells frequency and T cells infiltration into tumor, which led to tumor growth inhibition.

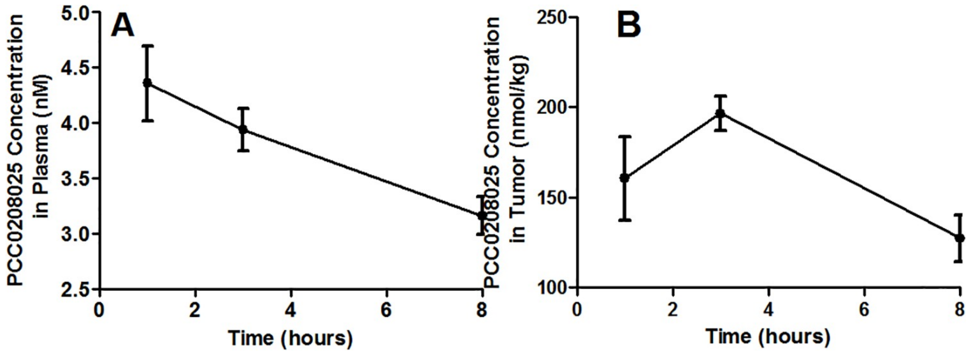

Fig 8. Pharmacokinetics of PCC0208025 in plasma and tumors in melanoma-bearing mice. After a single dose of 60 mg/kg PCC0208025, average concentrations were about 4.36, 3.94 and 3.16 nM in plasma at 1h, 3h and 8h, respectively (each time point, n = 5); and about 160.7, 196.7 and 127.3 nmol/kg in tumor at 1h, 3h and 8h, respectively (each time point, n = 5).

https://doi.org/10.1371/journal.pone.0228339.g008

A series of cytokines, such as IL-2, IFN-γ and TNF-α, can promote the CD8+ cytotoxic T cells production [2]. The activated CD8+ T cells into the tumor microenvironment can secrete the cytokine IFN-γ, which is closely related with the antitumor activity [24]. In our study, the results showed that PCC0208025 not only increased the IFN-γ level in plasma, but also increased the number of CD8+IFN-γ+ T cells in tumors in B16-F10 melanoma-bearing mice, which either induce immune cells proliferation and differentiation or produce direct tumor-killing effects to inhibit tumor growth [24].

In tumors, Treg are viewed as anti-tumor suppressors, and can decrease anti-tumor immune responses [25]. Most Treg cells are defined based on CD4, CD25 and FOXP3 expression. However, FOXP3 has a limited expression in Treg cells [25]. Recently, low level of CD127 on Treg cell surface are found, which is inversely correlated with FOXP3 level [25,26]. Thus, CD4+CD25+CD127$^{low/-}$ can be the more reliable biomarker of Treg cells. PD-1 antibodies can decrease the Treg level and prevent Treg-mediated inhibition of cytotoxic T lymphocytes [23,25,27]. In our study, PCC0208025 not only decreased the percentage of Treg (CD4+CD25+CD127$^{low/-}$ T cells), but also increased the ratios of CD8+/Treg in tumor. These results indicated that PCC0208025 inhibited Treg expansion and increased cytotoxic activity of tumor-infiltrating CD8+ T cells by the blockade of PD-1/PD-L1 binding.

Our pharmacokinetics study found that the average concentrations of PCC0208025 in plasma and tumor, at 1h, 3h and 8h after B16-F10 tumor-bearing mice were administrated with 60 mg/kg of PCC0208025, were similar to the IC_{50} values of PCC0208025 blockade against PD-1 and PD-L1 binding. These results indicated that PCC0208025 can be easily absorbed and distributed into the tumors to obtain the higher concentrations for producing the blockade effects against PD-1 and PD-L1 binding.

In summary, the PD-1/PD-L1 antibodies have already widely used for treatment of melanoma. However, these antibodies have some disadvantages such as the immunogenicity and immunotoxicities. In contrast, the small molecule compounds have good oral bioavailability without the immunogenicity and immunotoxicities. The small molecule interaction "hot spots" on PD-L1 surfaces suggest the approaches for the PD-1/PD-L1 antagonist drug discovery [13], which triggers further activities to discover small molecule inhibitors for PD-1/PD-L1 interactions with pharmacological effects. Our work suggests that PCC0208025 exhibited anti-tumor effects in B16-F10 tumor isograft model through inhibiting Treg expansion and increasing cytotoxic activity of tumor-infiltrating CD8+ T cells by the blockade of PD-1/PD-L1 binding, which provides the pharmacological basis to develop small molecule inhibitors for PD-1/PD-L1 interactions for PCC0208025 as a lead compound.

Supporting information

S1 Fig. LC-MS analysis from compound PCC0208025.
(PDF)

S2 Fig. The 1H spectra data of compound PCC0208025.
(PDF)

S3 Fig. Flow cytometry plots for each group.
(PDF)

S1 Table. The inhibitory effects of PCC0208025 against PD-1 and PD-L1 binding. In HTRF assay, Tag2-PD-1 (20 nM final), PCC0208025 (0.15, 0.46, 1.37, 4.12, 12.35, 37.04, 111.11, 333.33 and 1000 nM, final) or BMS-936559 (0.002, 0.006, 0.024, 0.10, 0.39, 1.56, 6.25, 25 and 100 nM final), and Tag1-PD-L1 (2 nM final) were designed. After pre-incubation, anti-Tag1-EuK and anti-Tag2-XL665 was added into the assay well. The signals (665 nm/620 nm

ratio) were obtained on Tecan M200 PRO. HTRF ratio = (OD665 nm/OD620 nm) × 10^4.
(DOCX)

S2 Table. Effects of PCC0208025 on IFN-γ secreted by $CD3^+$ cells *in vitro*. Human $CD3^+$ cells were plated in the 96-well plate with a density of 1 × 10^5 cells/well in 100 μL of DMEM containing 10% FBS. The 50 μL of aCD3 and aCD28 (final concentration of 1 μg/mL for each) were added into 96-well plate without or with 50 μL of human PD-L1 protein (final concentation of 10 nM). Then 50 μL of BMS-936559 or PCC0208025 solution with the final concentrations of 0.01, 0.1, 1 and 10 μM were added to the wells and cultured for 72 h. The supernatants were collected for detection of IFN-γ by using human IFN-γ ELISA Kit.
(DOCX)

S3 Table. The statitic results and p values for effects of PCC0208025 on IFN-γ level in $CD3^+$ cells in vitro.
(DOCX)

S4 Table. Effects of PCC0208025 on tumor volume in B16-F10 tumor isograft model. B16-F10 tumors mice were administrated by oral gavage with PCC0208025 at 30 mg/kg or 60 mg/kg, twice daily. On days 7, 9, 11, 14, 16, 18 and 20, tumor volumes were detemined.
(DOCX)

S5 Table. Effects of PCC0208025 on tumor weight in B16-F10 tumor isograft model. B16-F10 tumors mice were administrated by oral gavage with PCC0208025 at 30 mg/kg or 60 mg/kg, twice daily. On day 20, the tumors were removed and weighed.
(DOCX)

S6 Table. Effects of PCC0208025 on plasma IFN-γ level in B16-F10-bearing mice. Before all the mice with tumors were decapitated, the blood samples from orbital venous sinus were collected into tubes with heparin for plasma preparation. Plasma IFN-γ level was determined by using mice ELISA kit.
(DOCX)

S7 Table. Effects of PCC0208025 on T cell subsets from tumor in B16-F10-bearing mice. T cell subsets were counted by flow cytometry from tumors in B16-F10-bearing mice. The percentage of $CD3^+$, $CD3^+CD8^+$, $CD8^+IFN\text{-}\gamma^+$, $CD3^+CD4^+$, and $CD4^+CD25^+CD127^{low/-}$ T cells were presented.
(DOCX)

S8 Table. Pharmacokinetics of PCC0208025 in plasma and tumors in melanoma-bearing mice. After a single dose of 60 mg/kg PCC0208025, PCC0208025 concentrations were detected in plasma and tumors at 1h, 3h and 8h, respectively.
(DOCX)

Acknowledgments

We thank Shandong Luye Pharmaceutical Co. Ltd. for providing some experiments conditions including the laboratory.

Author Contributions

Conceptualization: Jingwei Tian.

Investigation: Guangying Du, Wenyan Wang, Haibo Zhu, Ning Li, Huijuan Zhao, Zhaoju Dong.

Methodology: Zhengping Hu, Pengfei Yu, Guangying Du, Wenyan Wang, Haibo Zhu, Ning Li, Huijuan Zhao, Zhaoju Dong.

Project administration: Zhengping Hu, Pengfei Yu, Guangying Du, Haibo Zhu, Ning Li, Zhaoju Dong.

Writing – original draft: Liang Ye, Jingwei Tian.

Writing – review & editing: Liang Ye, Jingwei Tian.

References

1. Crunkhorn S. Immunotherapy: Vaccine patch to treat melanoma. *Nat Rev Drug Discov* 2017; 17:18.
2. Hu Z, Ye L, Xing Y, Hu J, Xi T. Combined SEP and anti-PD-L1 antibody produces a synergistic antitumor effect in B16-F10 melanoma-bearing mice. *Sci Rep* 2018, 8: 217. https://doi.org/10.1038/s41598-017-18641-y PMID: 29317734
3. Scott LJ. Nivolumab: A Review in Advanced Melanoma. *Drugs* 2015; 75:1413–1424. https://doi.org/10.1007/s40265-015-0442-6 PMID: 26220912
4. Lee L, Gupta M, Sahasranaman S. Immune Checkpoint inhibitors: An introduction to the next-generation cancer immunotherapy. *J Clin Pharmacol* 2016; 56:157–169. https://doi.org/10.1002/jcph.591 PMID: 26183909
5. Zarganes-Tzitzikas T, Konstantinidou M, Gao Y, Krzemien D, Zak K, Dubin G, et al. Inhibitors of programmed cell death 1 (PD-1): a patent review (2010–2015). *Expert Opin Ther Pat* 2016; 26:973–977. https://doi.org/10.1080/13543776.2016.1206527 PMID: 27367741
6. Zhu Z, Liu W, Gotlieb V. The rapidly evolving therapies for advanced melanoma—Towards immunotherapy, molecular targeted therapy, and beyond. *Crit Rev Oncol Hematol* 2016; 99:91–99. https://doi.org/10.1016/j.critrevonc.2015.12.002 PMID: 26708040
7. Yamada H, Hida N, Satoh H, Yamagishi T, Hiroshima Y, Yoshii S, et al. Improved outcomes with pembrolizumab treatment in two cases of double cancer including non-small-cell lung cancer. *Anti-cancer drugs* 2018; 30:105–109.
8. Mahoney KM, Freeman GJ, McDermott DF. The Next Immune-Checkpoint Inhibitors: PD-1/PD-L1 Blockade in Melanoma. *Clin Ther* 2015; 37:764–782. https://doi.org/10.1016/j.clinthera.2015.02.018 PMID: 25823918
9. Magiera-Mularz K, Skalniak L, Zak KM, Musielak B, Rudzinska-Szostak E, Berlicki L, et al. Bioactive Macrocyclic Inhibitors of the PD-1/PD-L1 Immune Checkpoint. *Angew Chem Int Ed Engl* 2017; 56:13732–13735. https://doi.org/10.1002/anie.201707707 PMID: 28881104
10. Sasikumar PG, Ramachandra M. Small-Molecule Immune Checkpoint Inhibitors Targeting PD-1/PD-L1 and Other Emerging Checkpoint Pathways. *BioDrugs* 2018; 32: 481–497. https://doi.org/10.1007/s40259-018-0303-4 PMID: 30168070
11. Li K, Tian H. Development of small-molecule immune checkpoint inhibitors of PD-1/PD-L1 as a new therapeutic strategy for tumour immunotherapy. *J Drug Target* 2019; 3:244–256.
12. Skalniak L, Zak KM, Guzik K, Magiera K, Musielak B, Pachota M, et al. Small-molecule inhibitors of PD-1/PD-L1 immune checkpoint alleviate the PD-L1-induced exhaustion of T-cells. *Oncotarget* 2017; 8:72167–72181. https://doi.org/10.18632/oncotarget.20050 PMID: 29069777
13. Zak KM, Grudnik P, Guzik K, Zieba BJ, Musielak B, Domling A, et al. Structural basis for small molecule targeting of the programmed death ligand 1 (PD-L1). *Oncotarget* 2016; 7:30323–30335. https://doi.org/10.18632/oncotarget.8730 PMID: 27083005
14. Udhwani T, Mukherjee S, Sharma K, Sweta J, Khandekar N, et al. Design of PD-L1 inhibitors for lung cancer. *Bioinformation* 2019, 28: 139–150.
15. Weinmann H. Cancer Immunotherapy: Selected Targets and Small-Molecule Modulators. *ChemMedChem* 2016; 11:450–466. https://doi.org/10.1002/cmdc.201500566 PMID: 26836578
16. Chupak LS, Zheng X. Compounds useful as immunomodulators. Bristol-Myers Squibb Company. 2015, WO2015034820 A1.
17. Lv G, Sun D, Zhang J, Xie X, Wu X, Fang W, et al. Lx2-32c, a novel semi-synthetic taxane, exerts antitumor activity against prostate cancer cells in vitro and in vivo. *Acta Pharm Sin B* 2017; 7:52–58. https://doi.org/10.1016/j.apsb.2016.06.005 PMID: 28119808

18. Yang Y, Guan D, Lei L, Lu J, Liu JQ, Yang G, et al. H6, a novel hederagenin derivative, reverses multidrug resistance in vitro and in vivo. *Toxicol Appl Pharmacol* 2018; 341:98–105. https://doi.org/10.1016/j.taap.2018.01.015 PMID: 29408042
19. Ma YT, Yang Y, Cai P, Sun DY, Sanchez-Murcia PA, Zhang XY, et al. A Series of Enthalpically Optimized Docetaxel Analogues Exhibiting Enhanced Antitumor Activity and Water Solubility. *J Nat Prod* 2018; 81:524–533. https://doi.org/10.1021/acs.jnatprod.7b00857 PMID: 29359935
20. Zhang D, Xu Q, Wang N, Yang Y, Liu J, Yu G, et al. A complex micellar system co-delivering curcumin with doxorubicin against cardiotoxicity and tumor growth. *Int J Nanomedicine* 2018; 13:4549–4561. https://doi.org/10.2147/IJN.S170067 PMID: 30127606
21. Meng X, Du G, Ye L, Sun S, Liu Q, Wang H, et al. Combinatorial antitumor effects of indoleamine 2,3-dioxygenase inhibitor NLG919 and paclitaxel in a murine B16-F10 melanoma model. *Int J Immunopath Ph* 2017; 30:215–226.
22. Sun S, Du G, Xue J, Ma J, Ge M, Wang H, et al. PCC0208009 enhances the anti-tumor effects of temozolomide through direct inhibition and transcriptional regulation of indoleamine 2,3-dioxygenase in glioma models. *Int J Immunopathol Pharmacol* 2018; 32:2058738418787991.
23. Toor SM, Syed Khaja AS, Alkurd I, Elkord E. In-vitro effect of pembrolizumab on different T regulatory cell subsets. *Clin Exp Immunol* 2018; 191:189–197. https://doi.org/10.1111/cei.13060 PMID: 28963773
24. Kim HM, Lim J, Yoon YD, Ahn JM, Kang JS, Lee K, et al. Anti-tumor activity of ex vivo expanded cytokine-induced killer cells against human hepatocellular carcinoma. *Int Immunopharmacol* 2007; 7:1793–1801. https://doi.org/10.1016/j.intimp.2007.08.007 PMID: 17996690
25. Whiteside TL. FOXP3+ Treg as a therapeutic target for promoting anti-tumor immunity. *Expert Opin Ther Targets* 2018; 22: https://doi.org/10.1080/14728222.2018.1451514 PMID: 29532697
26. Islas-Vazquez L, Prado-Garcia H, Aguilar-Cazares D, Meneses-Flores M, Galicia-Velasco M, Romero-Garcia S, et al. LAP TGF-Beta Subset of CD4(+)CD25(+)CD127(-) Treg Cells is Increased and Overexpresses LAP TGF-Beta in Lung Adenocarcinoma Patients. *Biomed Res Int* 2015; 2015:430943.
27. Wang W, Lau R, Yu D, Zhu W, Korman A, Weber J. PD1 blockade reverses the suppression of melanoma antigen-specific CTL by CD4+ CD25(Hi) regulatory T cells. *Int Immunol* 2009; 21:1065–1077. https://doi.org/10.1093/intimm/dxp072 PMID: 19651643
